# Deep Learning Algorithms Versus Radiologists in Digital Breast Tomosynthesis for Breast Cancer Detection: Systematic Review and Meta-Analysis

**DOI:** 10.2196/91659

**Published:** 2026-05-06

**Authors:** Shewen Lyu, Zepeng Wang, Yujing Mu, Luyao Wang, Xiaohua Pei

**Affiliations:** 1 Beijing University of Chinese Medicine Third Affiliated Hospital, 51 Xiaoguan Street, Andingmenwai, Chaoyang District, Beijing, 100029, China, 86 13911683278

**Keywords:** digital breast tomosynthesis, deep learning, breast neoplasms, diagnostic accuracy, meta-analysis, artificial intelligence, AI

## Abstract

**Background:**

Deep learning (DL) algorithms for digital breast tomosynthesis (DBT) have proliferated, demonstrating emerging potential in enhancing lesion detection and classification.

**Objective:**

This study aimed to compare the diagnostic performance of DL algorithms for DBT with that of radiologists of varying experience and assess the clinical impact of DL assistance.

**Methods:**

A systematic search of PubMed, Embase, Web of Science, and the Cochrane Library was conducted up to November 8, 2025. Included studies compared the performance of stand-alone DL algorithms for DBT, radiologist interpretation alone, and DL-assisted diagnosis. Study quality was assessed using the Prediction Model Risk of Bias Assessment Tool+Artificial Intelligence (PROBAST+AI). Performance metrics were pooled using bivariate random effects and generalized linear mixed models.

**Results:**

A total of 13 studies with 38,565 patients were included in the final analysis. Stand-alone DL algorithms achieved a pooled sensitivity of 0.88 (95% CI 0.80-0.93), specificity of 0.74 (95% CI 0.59-0.85), and area under the receiver operating characteristic curve (AUC) of 0.89 (95% CI 0.86-0.92). While DL performance showed no statistically significant difference compared to all radiologists (AUC=0.89 vs 0.88; *P*=.64) or senior radiologists (AUC=0.89 vs 0.90; *P*=.48), DL demonstrated significantly superior sensitivity compared to junior radiologists (0.88 vs 0.76; *P*=.03). Notably, DL assistance did not statistically improve diagnostic metrics for radiologists across any experience level. Meta-regression identified validation methods as a significant source of heterogeneity.

**Conclusions:**

DL algorithms for DBT exhibited strong diagnostic proficiency and showed higher sensitivity than junior radiologists, suggesting their potential utility as adjunctive tools to help reduce oversight in less experienced settings. However, given that DL assistance did not significantly elevate overall human performance, current models act primarily as supplementary aids rather than definitive clinical tools. Future prospective multimodal studies are warranted to validate these findings and optimize clinical integration.

## Introduction

Breast cancer is the most commonly diagnosed cancer and the leading cause of cancer deaths among women worldwide, with an estimated 2.3 million new cases and 666,000 deaths occurring worldwide in 2022. This profound global burden underscores the urgency for early and accurate detection to improve prognosis and reduce the burden of invasive treatments [1,2]. While imaging serves as the cornerstone of screening, the transition from digital mammography to digital breast tomosynthesis (DBT) has revolutionized clinical practice. By providing quasi-3D volumetric data, DBT significantly alleviates the issue of tissue superposition, thereby increasing cancer detection rates and reducing unnecessary recall rates [3].

Despite these advantages, DBT introduces new challenges. The substantial increase in image volume significantly prolongs interpretation time, contributing to radiologist fatigue and potential cognitive overload [4,5]. Furthermore, despite overall improvements in detection rates, challenges regarding false positives (FPs) and false negatives (FNs) persist, with specific subtypes such as invasive lobular carcinoma prone to being overlooked [4,6]. The interpretation of DBT imagery is also heavily dependent on the radiologist’s experience, which increases subjectivity and the risk of misdiagnosis [7].

To address these diagnostic bottlenecks, deep learning (DL) algorithms for DBT have been increasingly developed, demonstrating emerging potential in enhancing lesion detection and classification. These algorithms aim to assist radiologists by extracting complex feature representations that may be imperceptible to the human eye [7]. However, results across the literature are inconsistent. While some pivotal studies suggest DL superiority [8], others indicate that algorithms may struggle with FPs or lack generalizability across different vendors and populations [9,10]. Moreover, the comparative performance of DL against radiologists of varying expertise (eg, junior and senior radiologists) remains a subject of ongoing debate [9,10]. This review includes data from 38,565 patients across the included studies.

Given the rapid accumulation of new evidence and the heterogeneity of study designs, a rigorous synthesis of current data is warranted. Therefore, the purpose of this systematic review and meta-analysis was to comprehensively compare the relative diagnostic performance and added value of DL algorithms vs radiologists of varying experience levels and evaluate potential factors influencing the diagnostic performance of these algorithms.

## Methods

The meta-analysis was carried out in full compliance with the PRISMA-DTA (Preferred Reporting Items for Systematic Reviews and Meta-Analyses extension for Diagnostic Test Accuracy) guidelines [11]. Furthermore, the protocol for this study was registered with PROSPERO (CRD420251242858).

### Ethical Considerations

This was a systematic review and meta-analysis, so ethics approval and consent to participate are not applicable.

### Search Strategy

A comprehensive literature search was conducted up to November 8, 2025, across 4 electronic databases: PubMed, Embase, Web of Science, and Cochrane Library. The search strategy, designed and executed to maximize sensitivity, used a combination of free-text terms and controlled vocabulary (eg, MeSH [Medical Subject Headings] terms in PubMed). Key concepts included three domains: (1) artificial intelligence (AI; eg, “Deep Learning”), (2) the target disease (eg, “Breast Neoplasms”), and (3) the imaging modality (eg, “Digital Breast Tomosynthesis”). No restrictions were placed on language, publication date, or study type. Two independent reviewers (SL and YM) performed initial title and abstract screening followed by full-text assessment of potentially eligible studies. To ensure literature saturation, the reference lists of all included articles were manually screened. The full, detailed search syntax for each database is provided in Table S1 in Multimedia Appendix 1.

### Inclusion and Exclusion Criteria

Studies were selected according to the participants, index test, target condition, reference standard, outcomes, and setting framework: women undergoing breast cancer screening via DBT (participants); evaluated the performance of stand-alone DL algorithms for DBT, independent radiologist interpretation, or DL-assisted radiologist diagnosis (index test); breast cancer, confirmed via histopathology for positive cases (target condition); final diagnosis based on histopathology (for positive cases) or clinical imaging follow-up (for negative cases; reference standard); primary outcomes, including diagnostic performance measures (sensitivity, specificity, and area under the receiver operating characteristic [ROC] curve [AUC]), and secondary outcomes, comprising clinical impact metrics (detection rate, positive predictive value [PPV], and recall rate; outcomes); and original studies using retrospective or prospective cohorts from screening programs or clinical databases (setting).

In addition, we systematically excluded studies whose titles and abstracts were clearly irrelevant, as well as noneligible publication types, including reviews, case reports, conference abstracts, meta-analyses, and letters to the editor. Furthermore, studies that did not involve DL for DBT; without algorithm comparison; and with true positive (TP), FP, true negative (TN), and FN data not available were also excluded. The screening process was conducted in duplicate by 2 independent reviewers (SL and ZW), with any disagreements resolved through consultation with a third reviewer (XP).

### Quality Assessment and Certainty of Evidence

We used the updated Prediction Model Risk of Bias Assessment Tool+AI (PROBAST+AI) quality assessment tool [12], which replaces the original 2019 Prediction Model Risk of Bias Assessment Tool instrument. This tool adopts a 2-phase structure comprising model development and model evaluation. Each phase includes 7 domains addressing participants, data sources, predictors, outcome assessment, and analytical approaches. For each domain, the risk-of-bias judgment is categorized as low, high, or unclear based on responses to predefined signaling questions. The full set of signaling questions and evaluation templates can be found in Tables S2 and S3 in Multimedia Appendix 1. To ensure objectivity and accuracy in the evaluation process, 2 reviewers (SL and LW) independently assessed the risk of bias in the included studies. The certainty of evidence was evaluated using the Grading of Recommendations Assessment, Development, and Evaluation (GRADE) framework [13]. Detailed evaluation items, decision rules, and domain-specific judgments can be found in Table S4 in Multimedia Appendix 1.

### Data Extraction

Two reviewers (SL and YM) independently extracted data from the included full-text articles, and disagreements were resolved through consultation with a third reviewer (XP). Extracted items included patient characteristics, details of DL methods, DBT imaging parameters, stand-alone DL performance, radiologist performance, and radiologist performance with DL assistance. Because most studies did not report full contingency tables, we used two strategies to derive TP, FP, FN, and TN values: (1) back calculation using reported sensitivity, specificity, number of positive cases, and total sample size; and (2) redigitizing ROC curves using the GetData software to obtain optimal sensitivity and specificity based on the Youden index. For DL algorithms, only validation set results were collected. When multiple DL models or radiologists were reported with overlapping cohorts, only the best-performing result (highest AUC) was extracted to avoid duplication.

### Outcome Measures

The primary outcome measures included the sensitivity, specificity, and AUC of DL algorithms, radiologists, and radiologists assisted by DL, as well as detection rate, PPV, and recall rate. Secondary outcomes focused on diagnostic performance stratified by radiologist experience both with and without DL assistance. Sensitivity (TP rate) reflected the ability to correctly identify cancer cases and was calculated as TP/(TP + FN) × 100%. Specificity (TN rate) represented the ability to correctly identify noncancer cases, calculated as TN/(TN + FP) × 100%. AUC summarizes overall discriminative ability. Detection rate was defined as TP/N × 100%, representing the proportion of cancers correctly detected in the screening population. PPV was calculated as TP/(TP + FP) × 100%, and recall rate was calculated as (TP + FP)/N × 100%. Radiologists with less than 5 years of experience were classified as junior, those with 5 or more years of experience were classified as senior, and studies without explicit experience data were categorized as unspecified.

### Statistical Analysis

Given the expected methodological heterogeneity across studies, we applied a bivariate random-effects model [14] to pool sensitivity, specificity, and AUC estimates. For PPV, recall rate, and detection rate, log-transformation was performed prior to synthesis using a random-effects generalized linear mixed model framework. Differences in pooled diagnostic performance were assessed using a mean *Z*-test, with statistical significance defined as a *P* value of less than .05. Heterogeneity was quantified using the Higgins *I*^2^ statistic [15]. For substantial heterogeneity (*I*^2^>50%), bivariate box plots were used to explore potential sources, and multivariable meta-regression was conducted for DL algorithms to evaluate the impact of validation strategy, study design, region of interest, and data splitting method. Temporal changes in DL performance were examined using bubble plots, whereas violin plots were used to visualize differences in radiologist performance before and after DL assistance. Fagan nomograms were generated to assess the clinical implications for patients. The assessment of publication bias was conducted using the Deeks funnel plot [16]. Analyses were executed using Stata (version 15.1; StataCorp) with the *midas* and *metadta* commands, as well as R (version 4.5.1; R Foundation for Statistical Computing) using the *ggplot2* and *tidyverse* packages.

## Results

### Literature Search and Study Selection

**Figure 1.**
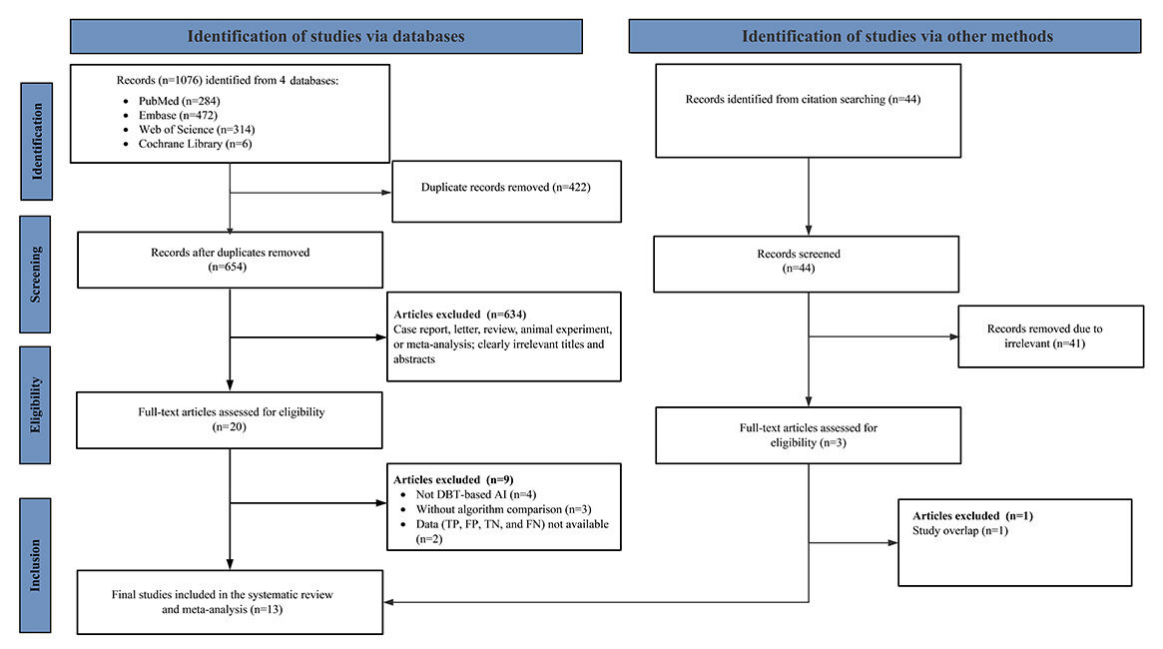
PRISMA (Preferred Reporting Items for Systematic Reviews and Meta-Analyses) flow diagram illustrating the study selection process. DBT: digital breast tomosynthesis; DL: deep learning; FN: false negative; FP: false positive; TN: true negative; TP: true positive.

A total of 1076 potentially relevant records were identified through the initial database search. Of these 1076 records, after removing 422 (39.2%) duplicates, 654 (60.8%) proceeded to title and abstract screening. During this stage, of the 654 remaining articles, 634 (96.9%) were excluded due to clear irrelevance or noneligible publication types. The remaining 20 articles were assessed in full text. Following detailed evaluation, of these 20 articles, 2 (10%) [17,18] were excluded because the data required to construct contingency tables (TP, FP, TN, and FN) were unavailable, 4 (20%) [19-22] were excluded because they were not research on DL for DBT, and 3 (15%) [23-25] lacked direct algorithm comparisons. In addition, 2 eligible records were identified from nondatabase sources. Ultimately, 13 studies met all the inclusion criteria and were incorporated into the meta-analysis [8-10,26-35]. The study selection process followed the PRISMA (Preferred Reporting Items for Systematic Reviews and Meta-Analyses) guidelines and is illustrated in Figure 1.

### Study Description and Quality Assessment

A total of 13 studies met the eligibility criteria. Among these, 46.2% (n=6) [8,9,28,31,32,35] reported internal validation cohorts (n=25,885 patients), and 61.5% (n=8) [9,10,26,27,29,30,33,34] reported external validation cohorts (n=12,680 patients; n=1, 7.7% of the studies reported both). In total, 61.5% (n=8) of the studies [9,10,26,27,29,30,33,34] evaluated stand-alone DL algorithms, and 84.6% (n=11) [8-10,26,28-33,35] assessed changes in radiologist performance before and after DL assistance. All studies (n=13, 100%) [8-10,26-35] reported outcomes for the overall radiologist group, whereas 15.4% (n=2) [10,35] included junior radiologists, 30.8% (n=4) [10,27,31,35] included senior radiologists, and 69.2% (n=9) [8,9,26,28-30,32-34] involved radiologists with unspecified experience levels. The included studies were published between 2017 and 2025. A total of 92.3% (n=12) [8-10,27-35] of the studies were retrospective in design, and 7.7% (n=1) [26] were prospective; all used pathological biopsy as the reference standard. Detailed characteristics are summarized in Tables S5 to S11 in Multimedia Appendix 1.

**Figure 2.**
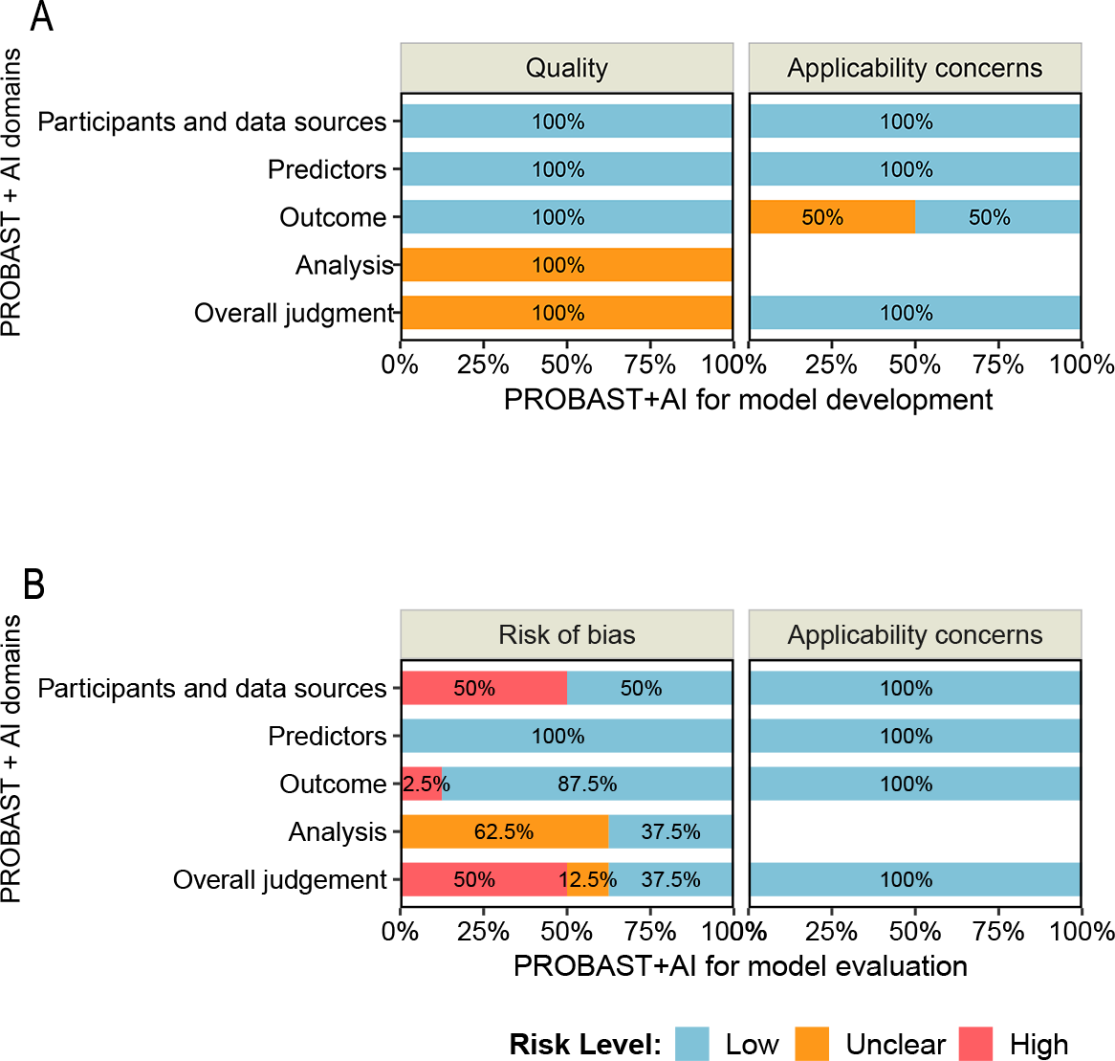
Risk of bias and applicability concerns of the included studies using the Prediction Model Risk of Bias Assessment Tool+Artificial Intelligence (PROBAST+AI) tool: (A) summary of PROBAST+AI assessment for model development and (B) summary of PROBAST+AI assessment for model evaluation.

The quality assessment using the PROBAST+AI tool is shown in Figure 2 and Tables S2 and S3 in Multimedia Appendix 1. For model development, no study was rated as high risk in terms of either overall risk of bias or applicability concerns. For model evaluation, 50% (4/8) of the studies were judged to have high overall risk of bias, whereas none showed high applicability concerns. Overall, although the risk of bias in the evaluation phase was notable, the applicability of the included studies was generally acceptable. According to the GRADE framework, the certainty of the evidence ranged from low to moderate, primarily downgraded due to risk of bias and imprecision, as detailed in Table S4 in Multimedia Appendix 1.

### DL Algorithms

**Figure 3.**
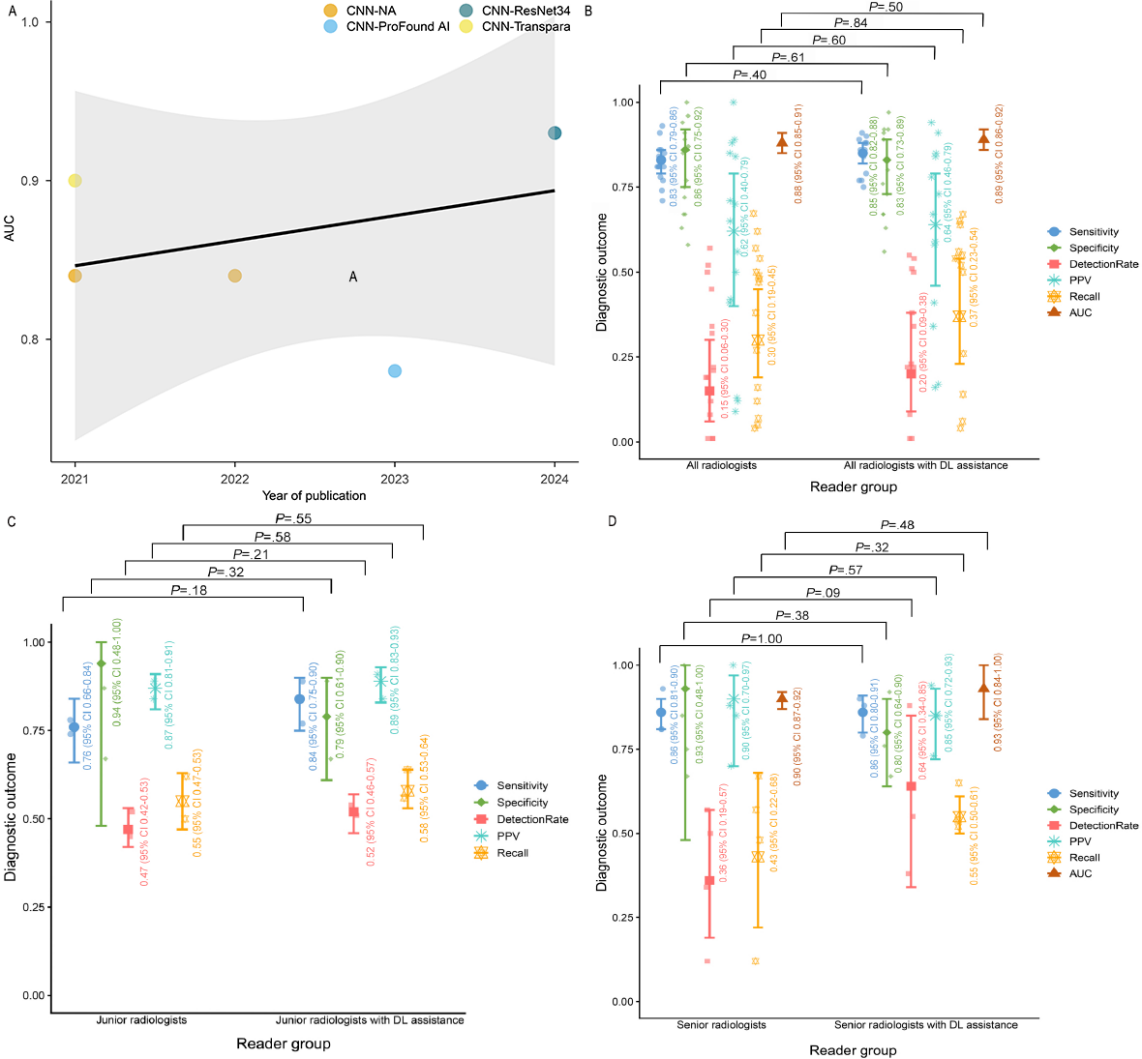
Bubble plot and violin plots: (A) bubble plot of temporal trends in area under the receiver operating characteristic curve (AUC) performance of different deep learning (DL) models, (B) violin plots of diagnostic outcomes for all radiologists before and after DL assistance, (C) violin plots of diagnostic outcomes for junior radiologists before and after DL assistance, and (D) violin plots of diagnostic outcomes for senior radiologists before and after DL assistance. AI: artificial intelligence; CNN: convolutional neural network; NA: not available; PPV: positive predictive value; ResNet: residual neural network.

The sensitivity of DL algorithms was found to be 0.88 (95% CI 0.80-0.93; *I*^2^=96.22%; low certainty), and the specificity was 0.74 (95% CI 0.59-0.85; *I*^2^=99.63%; low certainty). The AUC was 0.89 (95% CI 0.86-0.92; low certainty), whereas the detection rate was 0.14 (95% CI 0.06-0.29; *I*^2^=98.8%; low certainty). The PPV was 0.41 (95% CI 0.18-0.70; *I*^2^=99.1%; low certainty), and the recall rate was 0.39 (95% CI 0.27-0.53; *I*^2^=99.7%; low certainty). As shown in Tables1  2 and Figures S1 to S5 in Multimedia Appendix 1, the bubble chart indicates that the AUC values remained relatively stable from 2021 to 2024, as shown in Figure 3A.

**Table 1.** Deep learning (DL) algorithms for digital breast tomosynthesis (DBT) vs radiologists of different experience levels in terms of diagnostic performance outcomes for breast cancer diagnosis.

Subgroup	Sample size, n	Validation datasets, n	Sensitivity (95% CI)	Difference in sensitivity^a^		Specificity (95% CI)	Difference in specificity^a^		AUC^b^ (95% CI)	Difference in AUC^a^	
				*Z*-score	*P* value		*Z*-score	*P* value		*Z*-score	*P* value
Overall DL for DBT	12,555	8	0.88 (0.80-0.93)	—^c^	—	0.74 (0.59‐0.85)	—	—	0.89 (0.86-0.92)	—	—
DL for DBT internal validation	5182	1	0.96 (0.90-1.00)	2.63	.009	0.40 (0.03-0.76)	1.95	.05	0.84 (0.78-0.89)	1.62	.11
DL for DBT external validation	7373	7	0.85 (0.77-0.90)	—	—	0.78 (0.65-0.87)	—	—	0.89 (0.86-0.91)	—	—
DL for DBT vs all radiologists				1.33	.18		1.51	.13		0.46	.64
DL for DBT	12,555	8	0.88 (0.80-0.93)			0.74 (0.59-0.85)			0.89 (0.86-0.92)		
All radiologists	36,245	14	0.83 (0.79-0.86)			0.86 (0.75-0.92)			0.88 (0.85-0.91)		
DL for DBT vs junior radiologists				2.12	.03		1.35	.18		—	—
DL for DBT	12,555	8	0.88 (0.80-0.93)			0.74 (0.59-0.85)			0.89 (0.86-0.92)		
Junior radiologists	310	2	0.76 (0.66-0.84)			0.94 (0.48-1.00)			—		
DL for DBT vs senior radiologists				0.50	.62		1.28	.20		.50	.62
DL for DBT	12,555	8	0.88 (0.80-0.93)			0.74 (0.59-0.85)			0.89 (0.86-0.92)		
Senior radiologists	961	4	0.86 (0.81-0.90)			0.93 (0.48-1.00)			0.90 (0.87-0.92)		

a On the basis of 2-sided *Z*-test.

b AUC: area under the receiver operating characteristic curve.

c Not applicable.

**Table 2.** Deep learning (DL) for digital breast tomosynthesis (DBT) vs radiologists of different experience levels in terms of clinical impact outcomes for breast cancer diagnosis.

Subgroup	Sample size n	Validation datasets, n	Detection rate (95% CI)	Difference in detection rate^a^		PPV^b^ (95% CI)	Difference in PPV^a^		Recall rate (95% CI)	Difference in recall rate^a^	
				*Z*-score	*P* value		*Z*-score	*P* value		*Z*-score	*P* value
Overall DL for DBT	12,555	8	0.14 (0.06-0.29)	—^c^	—	0.41 (0.18-0.70)	—	—	0.39 (0.27-0.53)	—	—
DL for DBT internal validation	5182	1	0.09 (0.08-0.09)	0.87	.38	0.13 (0.12-0.15)	2.33	.02	0.63 (0.62-0.65)	4.04	<.001
DL for DBT external validation	7373	7	0.15 (0.06-0.33)	—	—	0.47 (0.19-0.76)	—	—	0.36 (0.24-0.50)	—	—
DL for DBT vs all radiologists				0.18	.91		1.27	.21		0.96	.34
DL for DBT	12,555	8	0.14 (0.06-0.29)			0.41 (0.18-0.70)			0.39 (0.27-0.53)		
All radiologists	36,245	14	0.15 (0.06-0.30)			0.62 (0.40-0.79)			0.30 (0.19-0.45)		
DL for DBT vs junior radiologists				5.07	>.99		3.41	<.001		2.05	.04
DL for DBT	12,555	8	0.14 (0.06-0.29)			0.41 (0.18-0.70)			0.39 (0.27-0.53)		
Junior radiologists	310	2	0.47 (0.42-0.53)			0.87 (0.81-0.91)			0.55 (0.47-0.63)		
DL for DBT vs senior radiologists				1.94	.05		3.28	.001		0.25	.81
DL for DBT	12,555	8	0.14 (0.06-0.29)			0.41 (0.18-0.70)			0.39 (0.27-0.53)		
Senior radiologists	961	4	0.36 (0.19-0.57)			0.90 (0.70-0.97)			0.43 (0.22-0.68)		

a On the basis of 2-sided *Z*-test.

b PPV: positive predictive value.

c Not applicable.

### All Radiologists

The sensitivity of all radiologists was found to be 0.83 (95% CI 0.79-0.86; *I*^2^=70.78%; low certainty), and the specificity was 0.86 (95% CI 0.75-0.92; *I*^2^=99.35%; low certainty). The AUC was 0.88 (95% CI 0.85-0.91; low certainty), whereas the detection rate was 0.15 (95% CI 0.06-0.30; *I*^2^=99.4%; moderate certainty). The PPV was 0.62 (95% CI 0.40-0.79; *I*^2^=98%; low certainty), and the recall rate was 0.30 (95% CI 0.19-0.45; *I*^2^=99.5%; low certainty). As shown in Table 1 and 2 and Figures S6 to S10 in Multimedia Appendix 1, there were no statistically significant differences between the performance of DL algorithms and that of all radiologists across all metrics.

### Radiologists With Different Levels of Experience

DL algorithms for DBT achieved markedly higher sensitivity than junior radiologists (0.88 vs 0.76; *Z*=2.12; *P*=.03). However, DL algorithms for DBT exhibited significantly lower PPV than junior radiologists (0.41 vs 0.87; *Z*=3.41; *P*<.001) and significantly lower recall rate than junior radiologists (0.39 vs 0.55; *Z*=2.05; *P*=.04). Additionally, DL algorithms for DBT had a significantly lower PPV than senior radiologists (0.41 vs 0.90; *Z*=3.28; *P*=.001). These findings are illustrated in Tables1  2.

### Changes Before and After DL Assistance

With DL assistance, there were no significant improvements in any of the outcome measures for all radiologists. Similarly, there were no significant improvements in any of the outcome measures for junior or senior radiologists. These findings are shown in Tables S12 and S13 in Multimedia Appendix 1.

### Heterogeneity Testing: Bivariate Box Plots and Meta-Regression

**Figure 4.**
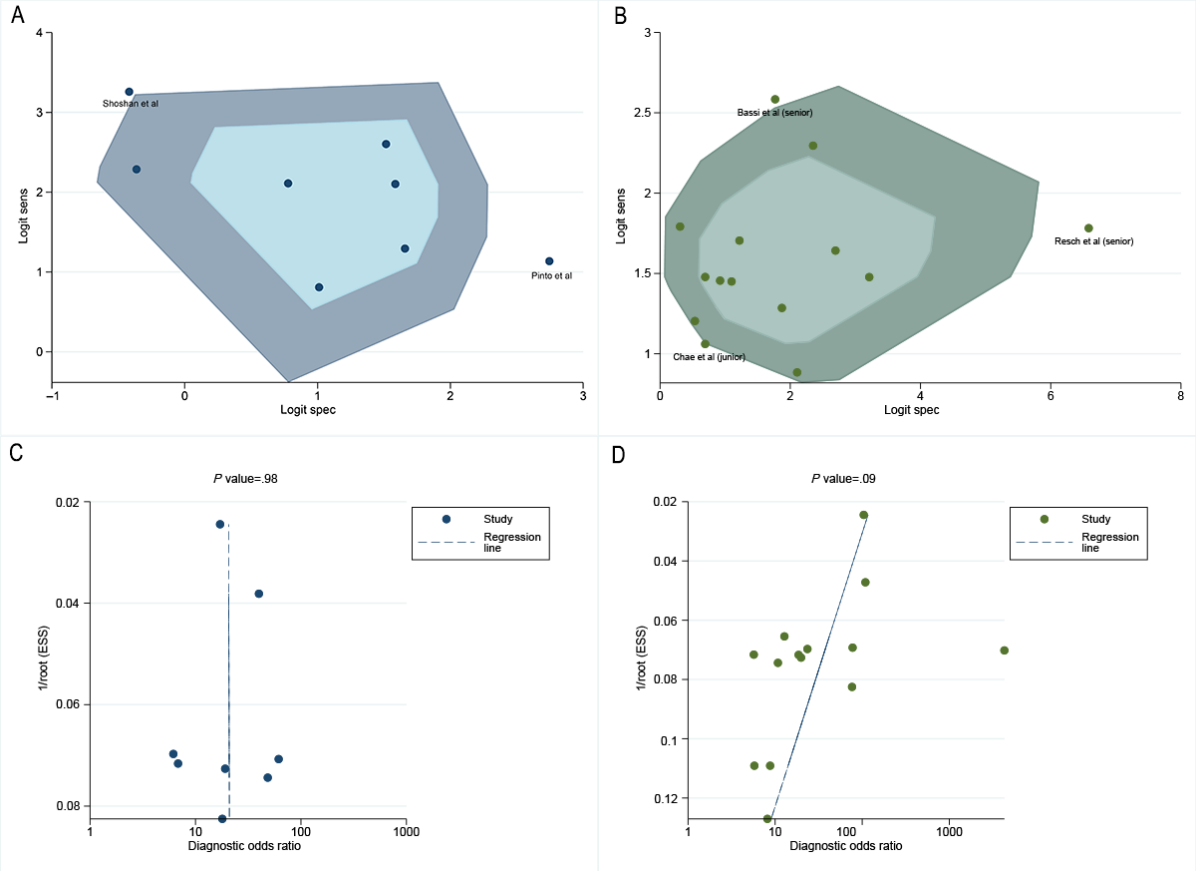
Bivariate box plots and Deeks funnel plots: (A) bivariate box plot for stand-alone deep learning (DL) for digital breast tomosynthesis (DBT) reading, (B) bivariate box plot for radiologist reading, (C) Deeks funnel plot for stand-alone DL for DBT reading, and (D) Deeks funnel plot for radiologist reading [9,10,27,29,35]. ESS: effective sample size.

The bivariate box plots suggested that the studies by Shoshan et al [9] and Pinto et al [29] might have contributed to the heterogeneity of DL algorithms, whereas those by Bassi et al [10] and Resch et al [27] might have been sources of heterogeneity among all radiologists, as shown in Figures 4A and 4B [9,10,27,29,35]. Meta-regression indicated that this heterogeneity primarily arose from differences in validation methods (internal validation vs external validation; specificity *P*=.05) and geographic regions (Europe vs North America; sensitivity *P*<.001), as shown in Table S14 in Multimedia Appendix 1.

### Sensitivity Analysis

To assess the robustness of the primary findings, 2 sensitivity analyses were conducted. First, after excluding outliers identified by the bivariate box plot graphical diagnostic method, the analysis yielded a sensitivity of 0.86 (95% CI 0.78‐0.91), specificity of 0.74 (95% CI 0.62‐0.83), AUC of 0.88 (95% CI 0.85‐0.91), detection rate of 0.13 (95% CI 0.05‐0.33), PPV of 0.38 (95% CI 0.14‐0.69), and recall of 0.36 (95% CI 0.23‐0.53). Second, after excluding studies assessed as having a high risk of bias using the PROBAST+AI tool in the validation set, the recalculated pooled effect sizes were as follows: sensitivity of 0.86 (95% CI 0.77‐0.91), specificity of 0.80 (95% CI 0.57‐0.93), AUC of 0.90 (95% CI 0.87‐0.92), detection rate of 0.21 (95% CI 0.15‐0.27), PPV of 0.61 (95% CI 0.37‐0.81), and recall of 0.40 (95% CI 0.26‐0.56). These results were consistent with those of the primary analysis, indicating that the overall conclusions regarding diagnostic performance were robust, neither unduly influenced by high-risk studies nor biased by individual outliers. These findings are shown in Table S15 in Multimedia Appendix 1.

### Clinical Application Value and Publication Bias

**Figure 5.**
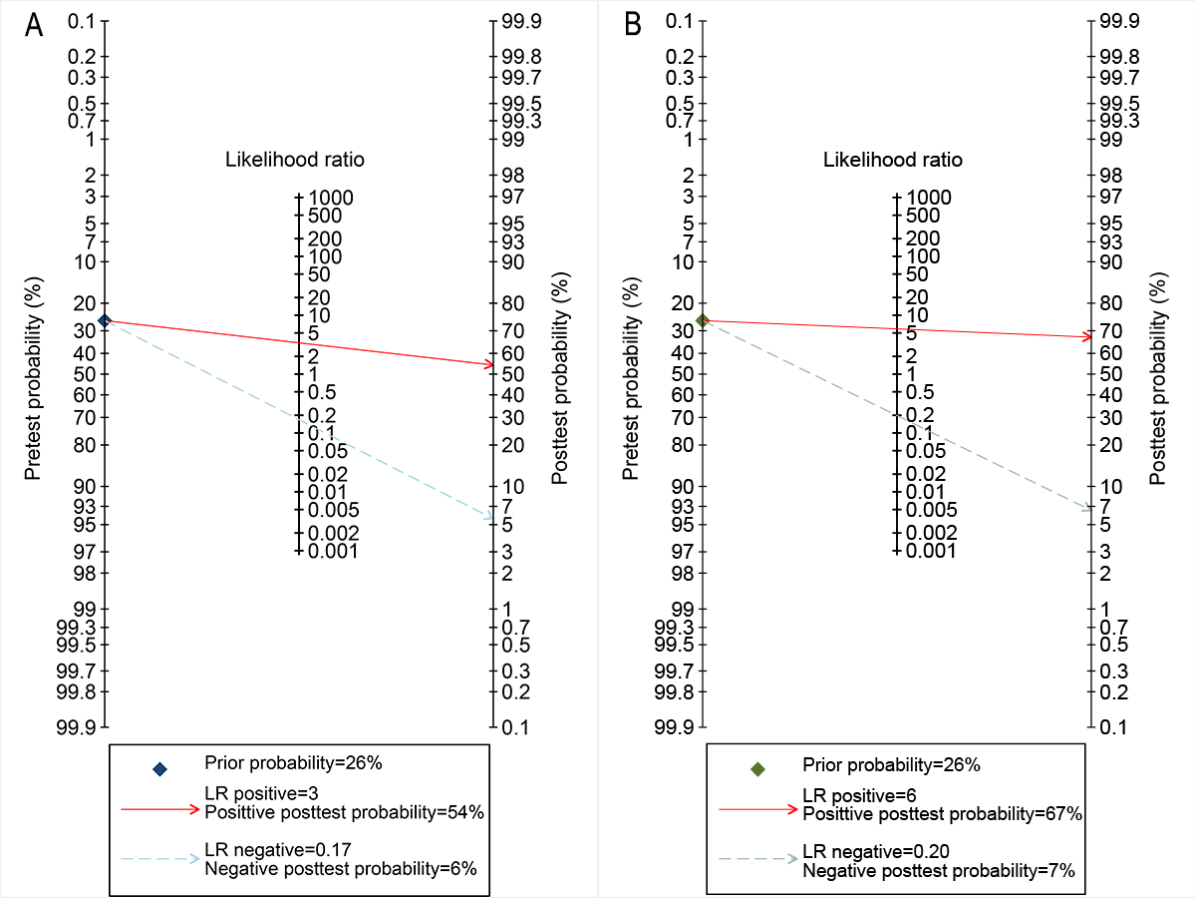
Fagan nomograms illustrating the posttest probability of breast cancer with digital breast tomosynthesis (DBT) reading: (A) stand-alone deep learning (DL) for DBT reading and (B) radiologist reading without DL assistance. LR: likelihood ratio.

The Deeks funnel plot asymmetry test showed no evidence of publication bias (0.98 vs 0.09), as illustrated in Figures 4C and 4D [9,10,27,29,35]. Using the median prevalence from the included studies as the prior probability, the Fagan nomogram for DL algorithms (median prevalence 26%, IQR 13.84%–38.95%) indicated a positive posttest probability of 54%, while that for all radiologists (median prevalence 26%, IQR 12.60%–55.01%) indicated a positive posttest probability of 67%, as shown in Figure 5.

## Discussion

### Principal Findings

Our systematic review and meta-analysis demonstrated that DL algorithms using DBT achieved diagnostic proficiency comparable to the aggregate performance of radiologists and senior experts while exhibiting significantly superior sensitivity compared to junior radiologists. The observed parity between DL and senior radiologists suggests that current computational models have attained a level of pattern recognition equivalent to seasoned clinical judgment. However, the fact that DL has not significantly superseded senior radiologists implies that, while algorithms facilitate standardization, they currently lack the nuance required to outperform complex human decision-making [36-38]. Conversely, the significant performance gap regarding junior practitioners likely stems from the algorithms’ robust capacity to systematically analyze vast volumes of annotated data, enabling the detection of subtle or occult lesions that may be obscured by the subjectivity or limited pattern recognition characteristic of early-career experience [39]. By minimizing the specific oversight errors associated with inexperience [40], DL models effectively function as a “safety net” enhancing diagnostic sensitivity. Consequently, these findings support the integration of DL not as a stand-alone replacement for expert review but as a vital adjunctive tool to augment the capabilities of less experienced radiologists and bridge diagnostic disparities in resource-constrained environments.

Regarding clinical impact metrics, our initial analysis showed that DL algorithms had lower PPV and recall rates than junior radiologists. However, these findings should be interpreted with caution as they likely reflect spectrum bias and the limited number of studies available for the junior subgroup (2/13, 15.4%). Specifically, the junior radiologist data were derived from highly enriched cohorts, with a malignancy prevalence of 70% in the study by Chae et al [35] and 57% positive findings in the study by Bassi et al [10]. As PPV is a prevalence-dependent metric, the exceptionally high disease burden in these datasets artificially inflated the pooled estimates for the junior subgroup, reflecting fundamental differences in the underlying test populations rather than intrinsic radiologist superiority.

More importantly, an essential clinical counterpoint to the sensitivity advantage of DL algorithms is the significantly lower pooled PPV of stand-alone DL algorithms (0.41) compared to all radiologists (0.62) and senior radiologists (0.90). A PPV of 0.41 implies that less than half of the cases flagged by the DL algorithms are truly malignant, corresponding to a substantial FP burden. This finding is particularly contradictory as one of the primary, well-documented advantages of DBT over traditional mammography is its ability to reduce unnecessary recall rates. Using low-PPV DL algorithms as a primary or concurrent reading tool could erode this advantage, leading to a cascade of adverse consequences. These include unnecessary biopsies, heightened patient psychological distress and anxiety, and the overconsumption of health care resources—costs that are thoroughly documented in breast screening literature. Therefore, we urge a cautious approach to claims advocating for current DL algorithms as universal standardization tools. Their integration should be highly context dependent: in settings experiencing a severe shortage of experienced radiologists, the sensitivity benefits may offset the FP costs; however, in fully resourced screening environments with available senior radiologists, the net clinical benefit of stand-alone DL remains uncertain. Before recommending these models as reliable standardization tools, future deployment frameworks must incorporate well-defined, acceptable safety thresholds for FP rates and prospectively evaluate downstream clinical and economic outcomes.

Interestingly, our analysis comparing radiologist performance with and without DL assistance revealed no statistically significant incremental benefit. This lack of synergistic enhancement likely results from a “ceiling effect,” where experienced radiologists and high-performing algorithms achieve similar diagnostic performance, leaving limited room for improvement [41,42]. Furthermore, the clinical utility of DL assistants is constrained by “automation bias” and the opaque nature of “black box” algorithms [7,43]. When radiologist confidence in the algorithm is low—or when the AI serves merely as a concurrent reader without explainable features—the translation of algorithmic output into improved decision-making is diminished [44]. This suggests that, for DL to provide substantive added value, future systems must move beyond binary classification to provide interpretability and context-aware insights.

In comparison with a previous meta-analysis published by Yoon et al [45] in 2023, which reported that stand-alone AI significantly outperformed radiologists (AUC=0.90 vs 0.79) for breast cancer diagnosis, our study presents more conservative findings (AUC=0.89 vs 0.88; no significant difference). This discrepancy is attributable to the substantial expansion of the evidence base in our review. By incorporating approximately 3 times the number of studies, our analysis mitigates small-study effects that may have overestimated AI superiority in previous reviews. Our analysis suggests that the diagnostic performance of DL algorithms is currently comparable with the aggregate performance of the overall radiologist population in DBT interpretation but not demonstrably superior.

Beyond prior analyses, we assessed the specific impact of the DL assistant by comparing diagnostic performance without and with the DL assistant, offering critical insights into the human-AI interaction. We also incorporated essential clinical impact metrics such as detection rate, PPV, and recall rate to assess practical utility beyond discrimination accuracy. Furthermore, the methodological rigor was strengthened through the application of the PROBAST+AI tool for quality assessment and the GRADE approach to evaluate the certainty of evidence. These enhancements and stratified analyses collectively supply new, high-quality evidence that clarifies the current application of DL in breast imaging.

The extreme heterogeneity observed in several pooled metrics (*I*^2^>98%) warrants careful interpretation. Although meta-regression identified the validation strategy (internal vs external) as a significant contributor to heterogeneity in specificity (*P*=.05), this single factor cannot account for the near-complete variance in metrics such as PPV and recall rate. We posit that the residual heterogeneity reflects fundamental differences inherent to the DL algorithms themselves. First, algorithm design thresholds vary considerably across vendors: some systems are deliberately calibrated toward high sensitivity for triage purposes [27], whereas others prioritize specificity to minimize FP recalls, resulting in divergent operating points along the ROC curve. Second, training set composition, including differences in malignancy prevalence, patient demographics, geographic regions, mammographic density distribution, and scanner hardware, introduces domain-specific biases that cannot be homogenized through statistical pooling alone [28]. Third, variability in FP tolerance thresholds across health care systems and screening programs further compounds interstudy differences [9]. Given this landscape, future studies should prioritize standardized reporting of operating thresholds and algorithm configurations to facilitate more meaningful comparisons.

Additionally, bivariate box plots pinpointed the studies by Shoshan et al [9] and Pinto et al [29] as distinct outliers. This heterogeneity is mechanistically explainable: Shoshan et al [9] optimized their algorithm for a “triage” workflow, deliberately sacrificing specificity to maximize sensitivity for ruling out normal cases, resulting in a skewed performance profile compared to standard diagnostic models. Meanwhile, the divergence in the study by Pinto et al [29] is attributable to a small sample size (N=190), which introduces substantial statistical instability and random variation into the results.

Looking ahead, most of the DL algorithms included in our study were confined to single-modality DBT data, lacking the contextual depth provided by supplementary imaging and clinical history. Future iterations must prioritize multimodal integration to synthesize comprehensive diagnostic insights by correlating findings with the patient’s clinical background [46]. Implementation is further constrained by technical and systemic barriers, including data scarcity, regulatory challenges, and limited generalizability. While emerging techniques such as few-shot learning and self-supervised models may address these gaps, sustained multidisciplinary efforts are essential to optimize AI safety and deliver comprehensive solutions that genuinely augment radiological practice [47]. Currently, the opacity of decision-making processes remains a critical hurdle, highlighting the urgent need for future AI systems to prioritize transparency and interpretability [48].

Some limitations of this meta-analysis should be considered when interpreting the results. First, the preponderance of retrospective designs introduces potential selection bias, necessitating validation through large-scale prospective trials. Second, to distinguish between independent datasets and mitigate the risk of patient overlap, we extracted data exclusively from the highest-performing DL algorithm in each study context. This methodological necessity may introduce an optimism bias, potentially leading to an overestimation of the average algorithmic performance. Third, the limited number of studies stratified by radiologist experience (particularly for juniors) constrains the statistical robustness of subgroup analyses. Fourth, because most included studies did not report complete contingency tables, we used the GetData software to redigitize published ROC curves and derived operating points using the Youden index. This approach introduces two layers of methodological limitation: (1) the manual redigitization process could introduce subjective measurement error, potentially introducing small but nonnegligible inaccuracies in the extracted sensitivity and specificity values; and (2) more critically, the Youden index–derived operating point represents a theoretical optimum rather than the actual clinical threshold used in each study’s real-world setting. Clinically deployed algorithms may operate at thresholds deliberately chosen to balance sensitivity and specificity according to institutional recall policies or regulatory requirements. Consequently, our extracted performance values may not faithfully reflect the true clinical performance of these algorithms as implemented, and this methodological limitation should be considered when interpreting the pooled estimates.

### Conclusions

In conclusion, DL algorithms for DBT demonstrated strong diagnostic performance. Although initial subgroup analyses indicated potentially higher sensitivity than junior radiologists, these findings are based on limited studies and require extensive validation to confirm their reliability. The current lack of a significant incremental benefit in human-AI collaborative workflows suggests that AI implementation should be approached cautiously—not as a stand-alone replacement or an automatic performance booster but rather as a supplementary “second opinion.” Future research must prioritize the development of explainable AI and prospective multimodal studies to better define the true synergistic potential of human-machine collaboration in breast cancer screening.

## Supplementary material

10.2196/91659 Multimedia Appendix 1 Supplementary tables and figures for the systematic review and meta-analysis.

10.2196/91659 Checklist 1 PRISMA checklist.

## Abbreviations

AI: artificial intelligence
AUC: area under the receiver operating characteristic curve
DBT: digital breast tomosynthesis
DL: deep learning
FN: false negative
FP: false positive
GRADE: Grading of Recommendations Assessment, Development, and Evaluation
MeSH: Medical Subject Headings
PPV: positive predictive value
PRISMA: Preferred Reporting Items for Systematic Reviews and Meta-Analyses
PRISMA-DTA: Preferred Reporting Items for Systematic Reviews and Meta-Analyses extension for Diagnostic Test Accuracy
PROBAST+AI: Prediction Model Risk of Bias Assessment Tool+Artificial Intelligence
ROC: receiver operating characteristic
TN: true negative
TP: true positive
