# Supplementary material for: Deep Learning Algorithms Versus Radiologists in Digital Breast Tomosynthesis for Breast Cancer Detection: Systematic Review and Meta-Analysis
Source: J Med Internet Res. 2026 May 6;28:e91659. doi: 10.2196/91659 (PMC13148330; doi:10.2196/91659)

**SUPPLEMENTARY MATERIALS**

**Deep Learning Algorithms versus Radiologists in Digital Breast Tomosynthesis for Breast Cancer Detection: A Systematic Review and Meta-Analysis**

**________________________________________________________**

**This supplementary document supports the main manuscript and provides detailed methodological descriptions, additional results, comprehensive search strategies, and quality assessment data.**

**Table of Contents**

**[Supplementary Table 1.](#_Toc225322967)** [Search strategy in PubMed, Embase, Cochrane and Web of Science. 4](#_Toc225322967)

**[Supplementary Table 2.](#_Toc225322968)** [Risk of bias assessment of model development using the PROBAST + AI. 7](#_Toc225322968)

**[Supplementary Table 3](#_Toc225322969)**[. Risk of bias assessment of model evaluation using PROBAST + AI. 9](#_Toc225322969)

**[Supplementary Table 4.](#_Toc225322970)** [Grading of recommendations, assessment, development, and evaluations (GRADE) scoring assessments in all the pooled outcomes. 11](#_Toc225322970)

**[Supplementary Table 5.](#_Toc225322971)** [Study and patient characteristics of the included studies. 12](#_Toc225322971)

**[Supplementary Table 6.](#_Toc225322972)** [Technical aspects information and diagnostic raw data of included studies for breast cancer diagnosis. 13](#_Toc225322972)

**[Supplementary Table 7.](#_Toc225322973)** [DL assistance subgroup analysis diagnostic data extraction. 14](#_Toc225322973)

**[Supplementary Table 8.](#_Toc225322974)** [Detection rate, recall rate, and PPV of the included studies. 15](#_Toc225322974)

**[Supplementary Table 9.](#_Toc225322975)** [Detection rate, recall rate, and PPV of the included studies for radiologists without DL assistance. 16](#_Toc225322975)

**[Supplementary Table 10.](#_Toc225322976)** [Detection rate, recall rate, and PPV of the included studies for radiologists with DL assistance. 17](#_Toc225322976)

**[Supplementary Table 11.](#_Toc225322977)** [Technical aspects of included studies for DBT. 18](#_Toc225322977)

**[Supplementary Table 12.](#_Toc225322978)** [DBT-based DL versus radiologists of different levels in the analysis of DBT-based artificial intelligence performance. 19](#_Toc225322978)

**[Supplementary Table 13.](#_Toc225322979)** [DBT-based DL versus radiologists of different levels in the analysis of DBT-based artificial intelligence performance. 20](#_Toc225322979)

**[Supplementary Table 14.](#_Toc225322980)** [Subgroup analysis and meta-regression analysis. 21](#_Toc225322980)

**[Supplementary Table 15](#_Toc225322981)**[. Sensitivity analysis. 21](#_Toc225322981)

**[Supplementary Figure 1.](#_Toc225322982)** [Forest plots of the pooled sensitivity and specificity of deep learning algorithms. 22](#_Toc225322982)

**[Supplementary Figure 2.](#_Toc225322983)** [Summary receiver operating characteristic (SROC) curve of deep learning algorithms. 23](#_Toc225322983)

**[Supplementary Figure 3.](#_Toc225322984)** [Forest plots of the pooled detection rate of deep learning algorithms. 24](#_Toc225322984)

**[Supplementary Figure 4.](#_Toc225322985)** [Forest plots of the pooled positive predictive value (PPV) of deep learning algorithms. 25](#_Toc225322985)

**[Supplementary Figure 5.](#_Toc225322986)** [Forest plots of the pooled recall rate of deep learning algorithms. 26](#_Toc225322986)

**[Supplementary Figure 6.](#_Toc225322987)** [Forest plots of the pooled sensitivity and specificity of all radiologists. 27](#_Toc225322987)

**[Supplementary Figure 7.](#_Toc225322988)** [Summary receiver operating characteristic (SROC) curve of all radiologists. 28](#_Toc225322988)

**[Supplementary Figure 8.](#_Toc225322989)** [Forest plots of the pooled detection rate of all radiologists. 29](#_Toc225322989)

**[Supplementary Figure 9.](#_Toc225322990)** [Forest plots of the pooled positive predictive value (PPV) of all radiologists. 30](#_Toc225322990)

**[Supplementary Figure 10.](#_Toc225322991)** [Forest plots of the pooled recall rate of all radiologists. 31](#_Toc225322991)

**[Supplementary Figure 11.](#_Toc225322992)** [Subgroup Analysis Forest plots of the sensitivity and specificity of senior radiologists vs junior radiologists. 32](#_Toc225322992)

**[Supplementary Figure 12](#_Toc225322993)**[. Summary receiver operating characteristic (SROC) curve of senior radiologists. 33](#_Toc225322993)

**[Supplementary Figure 13.](#_Toc225322994)** [Summary receiver operating characteristic (SROC) curve of all radiologists with DL assistance. 34](#_Toc225322994)

**[Supplementary Figure 14.](#_Toc225322995)** [Subgroup Analysis Forest plots of the sensitivity and specificity of radiologists with different levels of experience with DL assistance. 35](#_Toc225322995)

**[Supplementary Figure 15](#_Toc225322996)**[. Summary receiver operating characteristic (SROC) curve of senior radiologists with DL assistance. 36](#_Toc225322996)

**[Supplementary Figure 16.](#_Toc225322997)** [Forest plots of the pooled detection rate of all radiologists with DL assistance. 37](#_Toc225322997)

**[Supplementary Figure 17.](#_Toc225322998)** [Forest plots of the pooled positive predictive value (PPV) of all radiologists with DL assistance. 38](#_Toc225322998)

**[Supplementary Figure 18](#_Toc225322999)**[. Forest plots of the pooled recall rate of all radiologists with DL assistance. 39](#_Toc225322999)

### **Supplementary Table 1.** Search strategy in PubMed, Embase, Cochrane and Web of Science.

| **Database** | **Search strategy** | **Filters and Limits** | **Number of Studies** |
| --- | --- | --- | --- |
| PubMed | ("Breast Neoplasms"[Mesh] OR "breast cancer"[tiab] OR "breast cancers"[tiab] OR "breast carcinoma"[tiab] OR "breast carcinomas"[tiab] OR "breast neoplasm"[tiab] OR "breast neoplasms"[tiab] OR "breast malignancy"[tiab] OR "breast malignancies"[tiab] OR "breast tumor"[tiab] OR "breast tumors"[tiab] OR "breast tumour"[tiab] OR "breast tumours"[tiab] OR "breast mass"[tiab] OR "breast masses"[tiab] OR "breast lesion"[tiab] OR "breast lesions"[tiab])  AND  ("Artificial Intelligence"[Mesh] OR "Machine Learning"[Mesh] OR "Deep Learning"[tiab] OR "Radiomics"[Mesh] OR "Artificial Intelligence"[tiab] OR "AI"[tiab] OR "Computer Reasoning"[tiab] OR "Machine Intelligence"[tiab] OR "Computational Intelligence"[tiab] OR "Computer Vision Systems"[tiab] OR "Computer Vision System"[tiab] OR "Knowledge Acquisition"[tiab] OR "Knowledge Representations"[tiab] OR "Knowledge Representation"[tiab] OR "Hierarchical Learning"[tiab] OR "Machine Learning"[tiab] OR "Transfer Learning"[tiab] OR "Neural Networks, Computer"[tiab] OR "Artificial Neural Networks"[tiab] OR "radiomics"[tiab] OR "radiomic"[tiab] OR "texture analysis"[tiab] OR "quantitative imaging"[tiab] OR "computer-aided"[tiab] OR "CAD"[tiab])  AND  ("Digital Breast Tomosynthesis"[tiab] OR "DBT"[tiab] OR "breast tomosynthesis"[tiab] OR "tomosynthesis"[tiab] OR "3D mammograph*"[tiab] OR "three-dimensional mammograph*"[tiab] OR "3-D mammograph*"[tiab]) | No restrictions applied regarding date range, language, or publication type. | 284 |
| Embase | ('breast cancer'/exp OR 'breast cancer':ab,ti OR 'breast cancers':ab,ti OR 'breast carcinoma':ab,ti OR 'breast carcinomas':ab,ti OR 'breast neoplasm':ab,ti OR 'breast neoplasms':ab,ti OR 'breast malignancy':ab,ti OR 'breast malignancies':ab,ti OR 'breast tumor':ab,ti OR 'breast tumors':ab,ti OR 'breast tumour':ab,ti OR 'breast tumours':ab,ti OR 'breast mass':ab,ti OR 'breast masses':ab,ti OR 'breast lesion':ab,ti OR 'breast lesions':ab,ti)  AND  ('artificial intelligence'/exp OR 'machine learning'/exp OR 'deep learning'/exp OR 'deep learning':ab,ti OR 'radiomics'/exp OR 'artificial intelligence':ab,ti OR 'ai':ab,ti OR 'computer reasoning':ab,ti OR 'machine intelligence':ab,ti OR 'computational intelligence':ab,ti OR 'computer vision systems':ab,ti OR 'computer vision system':ab,ti OR 'knowledge acquisition':ab,ti OR 'knowledge representations':ab,ti OR 'knowledge representation':ab,ti OR 'hierarchical learning':ab,ti OR 'machine learning':ab,ti OR 'transfer learning':ab,ti OR 'neural networks, computer':ab,ti OR 'artificial neural networks':ab,ti OR 'radiomics':ab,ti OR 'radiomic':ab,ti OR 'texture analysis':ab,ti OR 'quantitative imaging':ab,ti OR 'computer-aided':ab,ti OR 'cad':ab,ti)  AND  ('digital breast tomosynthesis'/exp OR 'digital breast tomosynthesis':ab,ti OR 'dbt':ab,ti OR 'breast tomosynthesis':ab,ti OR 'tomosynthesis':ab,ti OR '3d mammograph*':ab,ti OR 'three-dimensional mammograph*':ab,ti OR '3-d mammograph*':ab,ti) | No restrictions applied regarding date range, language, or publication type. | 472 |
| Web of Science | TS=(("Breast Neoplasms" OR "breast cancer" OR "breast cancers" OR "breast carcinoma" OR "breast carcinomas" OR "breast neoplasm" OR "breast neoplasms" OR "breast malignancy" OR "breast malignancies" OR "breast tumor" OR "breast tumors" OR "breast tumour" OR "breast tumours" OR "breast mass" OR "breast masses" OR "breast lesion" OR "breast lesions"))  AND  TS=(("Artificial Intelligence" OR "Machine Learning" OR "Deep Learning" OR "Radiomics" OR "Artificial Intelligence" OR "AI" OR "Computer Reasoning" OR "Machine Intelligence" OR "Computational Intelligence" OR "Computer Vision Systems" OR "Computer Vision System" OR "Knowledge Acquisition" OR "Knowledge Representations" OR "Knowledge Representation" OR "Hierarchical Learning" OR "Machine Learning" OR "Transfer Learning" OR "Neural Networks, Computer" OR "Artificial Neural Networks" OR "radiomics" OR "radiomic" OR "texture analysis" OR "quantitative imaging" OR "computer-aided" OR "CAD"))  AND  TS=(("Digital Breast Tomosynthesis" OR "DBT" OR "breast tomosynthesis" OR "tomosynthesis" OR "3D mammograph*" OR "three-dimensional mammograph*" OR "3-D mammograph*")) | No restrictions applied regarding date range, language, or publication type. | 473 |
| Cochrane Library | ("Breast Neoplasms" OR "breast cancer" OR "breast cancers" OR "breast carcinoma" OR "breast carcinomas" OR "breast neoplasm" OR "breast neoplasms" OR "breast malignancy" OR "breast malignancies" OR "breast tumor" OR "breast tumors" OR "breast tumour" OR "breast tumours" OR "breast mass" OR "breast masses" OR "breast lesion" OR "breast lesions")  AND  ("Artificial Intelligence" OR "Machine Learning" OR "Deep Learning" OR "Radiomics" OR "AI" OR "Computer Reasoning" OR "Machine Intelligence" OR "Computational Intelligence" OR "Computer Vision Systems" OR "Computer Vision System" OR "Knowledge Acquisition" OR "Knowledge Representations" OR "Knowledge Representation" OR "Hierarchical Learning" OR "Transfer Learning" OR "Neural Networks, Computer" OR "Artificial Neural Networks" OR "radiomics" OR "radiomic" OR "texture analysis" OR "quantitative imaging" OR "computer-aided" OR "CAD")  AND  ("Digital Breast Tomosynthesis" OR "DBT" OR "breast tomosynthesis" OR "tomosynthesis" OR "3D mammography" OR "3D mammographies" OR "three-dimensional mammography" OR "three-dimensional mammographies" OR "3-D mammography" OR "3-D mammographies") | No restrictions applied regarding date range, language, or publication type. | 6 |

### **Supplementary Table 2.** Risk of bias assessment of model development using the PROBAST + AI.

| **Author, year** | | **Quality** | | | | **Applicability concerns** | | | **Overall judgement** | |
| --- | --- | --- | --- | --- | --- | --- | --- | --- | --- | --- |
|  |  | **Participants and data sources ^a^** | **Predictors ^b^** | **Outcome ^c^** | **Analysis ^d^** | **Participants and data sources ^e^** | **Predictors ^f^** | **Outcome ^g^** | **Quality ^h^** | **Applicability concerns ^i^** |
| Park et al. | 2024 | L | L | L | U | L | L | L | U | L |
| Shoshan et al. | 2022 | L | L | L | U | L | L | L | U | L |

**Abbreviation:** PROBAST+AI, Prediction model Risk of Bias Assessment Tool + AI, L low; H high; U unclear.

**Footnote:** Signaling questions are rated as "yes" (Y), "probably yes" (PY), "probably no" (PN), "no" (N), "no information" (NI), and in some cases "not applicable" (NA). All signaling questions are phrased in such a way that "yes" or "probably yes" indicates a low risk of bias. Any signaling questions rated as "no" or "probably no" indicate a potential high risk of bias in that domain. If there are no "no" or "probably no" ratings, but "no information" (NI) is present, the risk of bias in that domain is classified as unclear.

**a. Participants and data sources**

1.1 Were appropriate data sources used?

1.2 Was an appropriate study design used?

1.3 Did the in- and exclusions of study participants result in a representative dataset?

**b. Predictors**

2.1 Were predictors defined and assessed in a similar way for all participants?

2.2 Was any pre-processing of predictors similar for all participants?

2.3 Were predictor assessments made without knowledge of outcome data?

2.4 Were the predictors included in the model available at the time the model was intended to be used?

**c. Outcome**

3.1 Were outcomes defined and assessed appropriately?

3.2 Were outcomes defined and assessed in a similar way for all participants?

3.3 Were outcome assessments made without use or knowledge of predictor data?

3.4 Was the time interval between predictor assessment and outcome assessment appropriate?

**d. Analysis**

4.1 Was there evidence that the sample size was reasonable?

4.2 Were continuous and categorical predictors handled appropriately?

4.3 Were participants with missing or censored data handled appropriately in the analysis?

4.4 If methods to address class imbalance were used, was the model or the model predictions recalibrated?

4.5 Were methods used to address potential model overfitting?

**e. Participants and data sources**

Concern that the (data of the) included participants do not match the review question or the assessor’s intended use of the prediction model.

f. **Predictors**

Concern that the definition, pre-processing, assessment, or timing of assessment of the predictors in the model do not match the review question or the assessor’s intended use.

g. **Outcome**

Concern that the outcome, its definition, assessment, or timing of assessment do not match the review question or the assessor’s intended use.

**h. Quality**

Low risk: If all four domains were rated low concern regarding quality.

High risk: If at least one domain was rated high concern regarding quality .

Unclear: If at least one domain was rated unclear concern regarding quality and no domains were rated high concern.

**i. Applicability concerns**

Low risk: If all three domains were rated low concern for applicability.

High risk: If at least one domain was rated high concern for applicability.

Unclear: If at least one domain was rated unclear concern for applicability and no domains were rated high concern.

### **Supplementary Table 3**. Risk of bias assessment of model evaluation using PROBAST + AI.

| **Author, year** | | **Risk of bias** | | | | **Applicability concerns** | | | **Overall judgement** | |
| --- | --- | --- | --- | --- | --- | --- | --- | --- | --- | --- |
|  |  | **Participants and data sources ^a^** | **Predictors ^b^** | **Outcome ^c^** | **Analysis ^d^** | **Participants and data sources ^e^** | **Predictors ^f^** | **Outcome ^g^** | **Risk of bias ^h^** | **Applicability concerns ^i^** |
| Resch et al. | 2024 | H | L | H | U | L | L | L | H | L |
| Park et al. | 2024 | L | L | L | U | L | L | L | U | L |
| Chen et al. | 2025 | H | L | L | U | L | L | L | H | L |
| Winkel et al. | 2021 | L | L | L | L | L | L | L | L | L |
| Pinto et al. | 2021 | L | L | L | L | L | L | L | L | L |
| Bassi et al. | 2023 | H | L | L | U | L | L | L | H | L |
| Conant et al. | 2019 | L | L | L | L | L | L | L | L | L |
| Shoshan et al. | 2022 | H | L | L | U | L | L | L | H | L |

**Abbreviation:** PROBAST+AI, Prediction model Risk of Bias Assessment Tool + AI, L low; H high; U unclear.

**Footnote:** Signaling questions are rated as "yes" (Y), "probably yes" (PY), "probably no" (PN), "no" (N), "no information" (NI), and in some cases "not applicable" (NA). All signaling questions are phrased in such a way that "yes" or "probably yes" indicates a low risk of bias. Any signaling questions rated as "no" or "probably no" indicate a potential high risk of bias in that domain. If there are no "no" or "probably no" ratings, but "no information" (NI) is present, the risk of bias in that domain is classified as unclear.

**a. Participants and data sources**

1.1 Were appropriate data sources used?

1.2 Was an appropriate study design used?

1.3 Did the in- and exclusions of study participants result in a representative dataset?

**b. Predictors**

2.1 Were predictors defined and assessed in a similar way for all participants?

2.2 Was any pre-processing of predictors similar for all participants?

2.3 Were predictor assessments made without knowledge of outcome data?

2.4 Were the predictors included in the model available at the time the model was intended to be used?

**c. Outcome**

3.1 Were outcomes defined and assessed appropriately?

3.2 Were outcomes defined and assessed in a similar way for all participants?

3.3 Were outcome assessments made without use or knowledge of predictor data?

3.4 Was the time interval between predictor assessment and outcome assessment appropriate?

**d. Analysis**

4.1 Was model evaluation based on only apparent performance avoided?

4.2 Was there evidence that the sample size was reasonable?

4.3 Were participants with missing or censored data handled appropriately in the analysis?

4.4 If methods to address class imbalance were used, was the evaluation done in a dataset without imbalance correction?

4.5 If data splitting was done to create training and test datasets, was there evidence that data leakage was avoided?

4.6 If resampling methods were used to evaluate model performance, were all model development steps replicated in the resampling process?

4.7 Was the predictive performance of the model evaluated appropriately, e.g., calibration, discrimination, and net benefit?

**e. Participants and data sources**

Concern that the (data of the) included participants do not match the review question or the assessor’s intended use of the prediction model.

f. **Predictors**

Concern that the definition, pre-processing, assessment, or timing of assessment of the predictors in the model do not match the review question or the assessor’s intended use.

g. **Outcome**

Concern that the outcome, its definition, assessment, or timing of assessment do not match the review question or the assessor’s intended use.

**h. Risk of bias**

Low risk: If all four domains were rated low risk of bias.

High risk: If at least one domain was rated high risk of bias.

Unclear: If at least one domain was rated unclear risk of bias and no domains were rated high risk of bias.

**i. Applicability concerns**

Low risk: If all three domains were rated low concern for applicability.

High risk: If at least one domain was rated high concern for applicability.

Unclear: If at least one domain was rated unclear concern for applicability and no domains were rated high concern.

### **Supplementary Table 4.** Grading of recommendations, assessment, development, and evaluations (GRADE) scoring assessments in all the pooled outcomes.

| **Intervention** | **Outcome** | **Risk of Bias^a^** | **Inconsistency^b^** | **Indirectness^c^** | **Imprecision^d^** | **Publication Bias^e^** | **Total Downgrade** | **Final Rating** |
| --- | --- | --- | --- | --- | --- | --- | --- | --- |
| DBT-based DL | Sensitivity | 1 | 0 | 0 | 1 | 0 | 2 | Low |
|  | Specificity | 1 | 0 | 0 | 1 | 0 | 2 | Low |
|  | AUC | 1 | 0 | 0 | 1 | 0 | 2 | Low |
|  | Detection rates | 1 | 0 | 0 | 1 | 0 | 2 | Low |
|  | Recall | 1 | 0 | 0 | 1 | 0 | 2 | Low |
|  | PPV | 1 | 0 | 0 | 1 | 0 | 2 | Low |
|  |  |  |  |  |  |  |  |  |
| All radiologists | Sensitivity | 1 | 0 | 0 | 1 | 0 | 2 | Low |
|  | Specificity | 1 | 0 | 0 | 1 | 0 | 2 | Low |
|  | AUC | 1 | 0 | 0 | 1 | 0 | 2 | Low |
|  | Detection rates | 1 | 0 | 0 | 0 | 0 | 1 | Moderate |
|  | Recall | 1 | 0 | 0 | 1 | 0 | 2 | Low |
|  | PPV | 1 | 0 | 0 | 1 | 0 | 2 | Low |

a. **Risk of Bias:** Assessed using the PROBAST+AI tool. Downgraded by 1 level if at least one study had a high risk of bias.

b. **Inconsistency:** Assessed by the I² statistic. Downgraded by 1 level if I² > 50% and the source of heterogeneity could not be explained. No downgrade was applied if the source was identified.

c. **Indirectness (Population):** Downgraded by one level due to indirectness in the study population. The evidence was derived from a population that differs from the target population of interest.

d. **Imprecision:** Downgraded by 1 level if: for sensitivity, specificity, and AUC, the 95% CI width was > 0.20 or CIs crossed a clinical threshold (e.g., 0.80); for recall/detection rates/PPV, the 95% CI width was > 0.20; or if the total sample size was < 500 or positive/negative cases were < 100.

e. **Publication Bias:** Assessed using Deek's funnel plot asymmetry test. Downgraded by 1 level if the *P*-value was < 0.05.

### **Supplementary Table 5.** Study and patient characteristics of the included studies.

| **Author** | **Year** | **Country** | **Study design** | **Analysis** | **Reference standard** | **Density** | **No. of total sample size** | | | **No. of malignant breast cancer sample size** |
| --- | --- | --- | --- | --- | --- | --- | --- | --- | --- | --- |
|  |  |  |  |  |  |  | **Training** | **Internal**  **validation** | **External**  **validation** |  |
| Resch et al. | 2024 | Austria | Retro | PB | Pathology with clinical follow-up | Fatty: 84; Scattered: 154; Heterogeneous: 130; Extremely dense: 51 | NA | NA | 419 | External validation: 58 |
| Raya-Poveda et al. | 2021 | Spain | Retro | PB | Pathology with clinical follow-up | NA | NA | 15987 | NA | Internal validation: 113 |
| Park et al. | 2024 | Multi-countries | Retro | PB | Pathology with clinical follow-up | NA | 9455 | NA | 1355 | Training: 2866; External validation: 202 |
| Chen et al. | 2025 | USA | Retro | PB | Pathology with clinical follow-up | Fatty: 369; Scattered: 1417; Heterogeneous: 734; Extremely dense: 194; Not reported: 2110 | NA | NA | 4824 | External validation: 37 |
| Chae et al. | 2019 | Korea | Retro | PB | Pathology with clinical follow-up | Fatty: 3; Scattered: 15; Heterogeneous:48; Extremely dense: 34 | NA | 100 | NA | Internal validation: 70 |
| Kim et al. | 2024 | USA | Retro | PB | Pathology with clinical follow-up | Dense: 120; Nondense:120 | NA | 240 | NA | Internal validation: 100 |
| Winkel et al. | 2021 | USA | Pro | PB | Pathology with clinical follow-up | Fatty: 27; Scattered: 93; Heterogeneous:105; Extremely dense: 15 | NA | NA | 240 | External validation: 65 |
| Elías-Cabot et al. | 2024 | Spain | Retro | PB | Pathology with clinical follow-up | NA | NA | 6949 | NA | NA |
| Pinto et al. | 2021 | Netherlands | Retro | PB | Pathology with clinical follow-up | Fatty: 12; Scattered: 93; Heterogeneous:65; Extremely dense: 20 | NA | NA | 190 | External validation: 74 |
| Balleyguier et al. | 2017 | Multi-countries | Retro | PB | Pathology with clinical follow-up | Fatty: 5; Scattered: 35; Heterogeneous:33; Extremely dense: 7 | NA | 80 | NA | Internal validation: 21 |
| Bassi et al. | 2023 | Italy | Retro | PB | Pathology with clinical follow-up | Fatty: 19; Scattered: 70; Heterogeneous:111; Extremely dense: 10 | NA | NA | 210 | External validation: 120 |
| Conant et al. | 2019 | USA | Retro | PB | Pathology with clinical follow-up | Fatty/Scattered: 133; Heterogeneous/Extremely dense: 127 | NA | NA | 260 | External validation: 65 |
| Shoshan et al. | 2022 | USA | Retro | PB | Pathology with clinical follow-up | Fatty: 299; Scattered: 1756; Heterogeneous: 2714; Extremely dense: 370; Unknown: 43 | 5595 | 2529 | 5182 | Training: 831; Internal validation:182; External validation: 459 |

Retro retrospective; Pro prospective; PB patient-based; NA not available.

### **Supplementary Table 6.** Technical aspects information and diagnostic raw data of included studies for breast cancer diagnosis.

| **Author** | **Year** | **Data source** | **AI model** | **Data splitting method** | **AI method** | **AI algorithms ^a^** | **Internal validation** | | | | | **External validation** | | | | |
| --- | --- | --- | --- | --- | --- | --- | --- | --- | --- | --- | --- | --- | --- | --- | --- | --- |
|  |  |  |  |  |  |  | **TP** | **FP** | **FN** | **TN** | **AUC** | **TP** | **FP** | **FN** | **TN** | **AUC** |
| Resch et al. | 2024 | Local hospital | Image-only model | Hold-out | DL | CNN | NA | NA | NA | NA | NA | 54 | 65 | 4 | 296 | 0.93 |
| Raya-Povedano et al. | 2021 | Local hospital | Image-only model | Independent dataset validation | DL | CNN | NA | NA | NA | NA | NA | NA | NA | NA | NA | NA |
| Park et al. | 2024 | Local hospital | Image-only model | Independent dataset validation | DL | CNN | NA | NA | NA | NA | NA | 180 | 196 | 22 | 957 | 0.93 |
| Chen et al. | 2025 | Local hospital | Image-only model | Independent dataset validation | DL | CNN | NA | NA | NA | NA | NA | 33 | 1506 | 4 | 3281 | NA |
| Chae et al. | 2019 | Local hospital | Image-only model | Train/Validation/Test+K-fold CV | DL | CNN | NA | NA | NA | NA | NA | NA | NA | NA | NA | NA |
| Kim et al. | 2024 | Local hospital | Image-only model | Independent dataset validation | DL | CNN | NA | NA | NA | NA | NA | NA | NA | NA | NA | NA |
| Winkel et al. | 2021 | Local hospital | Image-only model | Independent dataset validation | DL | CNN | NA | NA | NA | NA | NA | 51 | 28 | 14 | 147 | 0.84 |
| Elías-Cabot et al. | 2024 | Local hospital | Image-only model | Independent dataset validation | DL | CNN | NA | NA | NA | NA | NA | NA | NA | NA | NA | NA |
| Pinto et al. | 2021 | Local hospital | Image-only model | Independent dataset validation | DL | CNN | NA | NA | NA | NA | NA | 56 | 7 | 18 | 109 | 0.90 |
| Balleyguier et al. | 2017 | Local hospital | Image-only model | Independent dataset validation | DL | CNN | NA | NA | NA | NA | NA | NA | NA | NA | NA | NA |
| Bassi et al. | 2023 | Local hospital | Image-only model | Independent dataset validation | DL | CNN | NA | NA | NA | NA | NA | 83 | 24 | 37 | 66 | 0.78 |
| Conant et al. | 2019 | Local hospital | Image-only model | Independent dataset validation | DL | CNN | NA | NA | NA | NA | NA | 59 | 115 | 6 | 80 | NA |
| Shoshan et al. | 2022 | Local hospital | Image&clinical model | Train/Validation/Test | DL | CNN | 442 | 2848 | 17 | 1875 | 0.84 | NA | NA | NA | NA | NA |

DL Deep learning; CNN Convolutional Neural Network; AUC Area Under the Curve; TP true positive; TN true negative; FP false positive; FN false negative; NA not available.

^a^ The optimal means the highest AUC value of the included study.

### **Supplementary Table 7.** DL assistance subgroup analysis diagnostic data extraction.

| **Author** | **Year** | **Without DL assistance** | | | | | | | | | | | | **With DL assistance** | | | | | | | | | | | |
| --- | --- | --- | --- | --- | --- | --- | --- | --- | --- | --- | --- | --- | --- | --- | --- | --- | --- | --- | --- | --- | --- | --- | --- | --- | --- |
|  |  | **Unspecified Radiologists** | | | | **Junior radiologists** | | | | **Senior radiologists** | | | | **Unspecified Radiologists** | | | | **Junior radiologists** | | | | **Senior radiologists** | | | |
|  |  | **TP** | **FP** | **FN** | **TN** | **TP** | **FP** | **FN** | **TN** | **TP** | **FP** | **FN** | **TN** | **TP** | **FP** | **FN** | **TN** | **TP** | **FP** | **FN** | **TN** | **TP** | **FP** | **FN** | **TN** |
| Raya-Poveda et al. | 2021 | 92 | 614 | 21 | 15260 | NA | NA | NA | NA | NA | NA | NA | NA | 95 | 493 | 18 | 15381 | NA | NA | NA | NA | NA | NA | NA | NA |
| Park et al. | 2024 | 55 | 44 | 10 | 149 | NA | NA | NA | NA | NA | NA | NA | NA | 57 | 39 | 8 | 154 | NA | NA | NA | NA | NA | NA | NA | NA |
| Chae et al. | 2019 | NA | NA | NA | NA | 52 | 10 | 18 | 20 | 57 | 10 | 13 | 20 | NA | NA | NA | NA | 54 | 10 | 16 | 20 | 55 | 10 | 15 | 20 |
| Kim et al. | 2024 | NA | NA | NA | NA | NA | NA | NA | NA | 81 | 35 | 19 | 105 | NA | NA | NA | NA | NA | NA | NA | NA | 90 | 34 | 10 | 106 |
| Winkel et al. | 2021 | 46 | 19 | 19 | 156 | NA | NA | NA | NA | NA | NA | NA | NA | 49 | 14 | 16 | 161 | NA | NA | NA | NA | NA | NA | NA | NA |
| Elias-Cabot et al. | 2024 | 40 | 300 | NA | NA | NA | NA | NA | NA | NA | NA | NA | NA | 67 | 329 | NA | NA | NA | NA | NA | NA | NA | NA | NA | NA |
| Pinto et al. | 2021 | 60 | 33 | 14 | 83 | NA | NA | NA | NA | NA | NA | NA | NA | 64 | 31 | 10 | 85 | NA | NA | NA | NA | NA | NA | NA | NA |
| Balleyguier et al. | 2017 | 18 | 25 | 3 | 34 | NA | NA | NA | NA | NA | NA | NA | NA | 18 | 26 | 3 | 33 | NA | NA | NA | NA | NA | NA | NA | NA |
| Bassi et al. | 2023 | NA | NA | NA | NA | 94 | 12 | 26 | 78 | 106 | 14 | 8 | 82 | NA | NA | NA | NA | 107 | 10 | 13 | 80 | 106 | 7 | 14 | 83 |
| Conant et al. | 2019 | 50 | 72 | 15 | 123 | NA | NA | NA | NA | NA | NA | NA | NA | 55 | 59 | 10 | 137 | NA | NA | NA | NA | NA | NA | NA | NA |
| Shoshan et al. | 2022 | 417 | 411 | 42 | 4312 | NA | NA | NA | NA | NA | NA | NA | NA | 413 | 302 | 46 | 4421 | NA | NA | NA | NA | NA | NA | NA | NA |

DL Deep learning; TP true positive; TN true negative; FP false positive; FN false positive; NA not available.

### **Supplementary Table 8.** Detection rate, recall rate, and PPV of the included studies.

| **Author** | **Year** | **Internal validation for DBT-based DL** | | | **External validation for DBT-based DL** | | |
| --- | --- | --- | --- | --- | --- | --- | --- |
|  |  | **Detection rate** | **Recall rate** | **PPV** | **Detection rate** | **Recall rate** | **PPV** |
| Resch et al. | 2024 | NA | NA | NA | 0.18 (54/296) | 0.40(119/296) | 0.45(54/119) |
| Raya-Povedano et al. | 2021 | NA | NA | NA | NA | NA | NA |
| Park et al. | 2024 | NA | NA | NA | 0.13(180/1355) | 0.28(376/1355) | 0.48(180/376) |
| Chen et al. | 2025 | NA | NA | NA | 0.01(33/4824) | 0.12(585/4824) | 0.02(33/1539) |
| Chae et al. | 2019 | NA | NA | NA | NA | NA | NA |
| Kim et al. | 2024 | NA | NA | NA | NA | NA | NA |
| Winkel et al. | 2021 | NA | NA | NA | 0.22(51/240) | 0.33(79/240) | 0.65(51/79) |
| Elías-Cabot et al. | 2024 | NA | NA | NA | NA | NA | NA |
| Pinto et al. | 2021 | NA | NA | NA | 0.30(56/190) | 0.33(63/190) | 0.89(56/63) |
| Balleyguier et al. | 2017 | NA | NA | NA | NA | NA | NA |
| Bassi et al. | 2023 | NA | NA | NA | 0.40(83/210) | 0.51(107/210) | 0.78(83/107) |
| Conant et al. | 2019 | NA | NA | NA | 0.23(59/260) | 0.67(174/260) | 0.34(59/174) |
| Shoshan et al. | 2022 | 0.09(442/5182) | 0.63(3290/5182) | 0.13(442/3290) | NA | NA | NA |

DL Deep learning; NA not available; PPV positive predictive value.

### **Supplementary Table 9.** Detection rate, recall rate, and PPV of the included studies for radiologists without DL assistance.

| **Author** | **Year** | **Unspecified radiologists** | | | **Junior radiologists** | | | **Senior radiologists** | | |
| --- | --- | --- | --- | --- | --- | --- | --- | --- | --- | --- |
|  |  | **Detection rate** | **Recall rate** | **PPV** | **Detection rate** | **Recall rate** | **PPV** | **Detection rate** | **Recall rate** | **PPV** |
| Resch et al. | 2024 | NA | NA | NA | NA | NA | NA | 0.12(50/411) | 0.12(50/411) | 1.00(50/50) |
| Raya-Povedano et al. | 2021 | 0.01(92/15987) | 0.04 (706/15 987) | 0.13(92/706) | NA | NA | NA | NA | NA | NA |
| Park et al. | 2024 | 0.21(55/258) | 0.38(99/258) | 0.56(55/99) | NA | NA | NA | NA | NA | NA |
| Chen et al. | 2025 | 0.01(31/4824) | 0.07(334/4824) | 0.09(31/334) | NA | NA | NA | NA | NA | NA |
| Chae et al. | 2019 | NA | NA | NA | 0.52(52/100) | 0.62(62/100) | 0.84(52/62) | 0.57(57/100) | 0.67(67/100) | 0.85(57/67) |
| Kim et al. | 2024 | NA | NA | NA | NA | NA | NA | 0.34(81/240) | 0.48(116/240) | 0.70(81/116) |
| Winkel et al. | 2021 | 0.19(46/240) | 0.27(65/240) | 0.71(46/65) | NA | NA | NA | NA | NA | NA |
| Elías-Cabot et al. | 2024 | 0.01(40/6953) | 0.05(340/6953) | 0.12(40/340) | NA | NA | NA | NA | NA | NA |
| Pinto et al. | 2021 | 0.32(60/190) | 0.49(93/190) | 0.65(60/93) | NA | NA | NA | NA | NA | NA |
| Balleyguier et al. | 2017 | 0.23(18/80) | 0.54(43/80) | 0.42(18/43) | NA | NA | NA | NA | NA | NA |
| Bassi et al. | 2023 | NA | NA | NA | 0.45(94/210) | 0.50(106/210) | 0.89(94/106) | 0.50(106/210) | 0.57(120/210) | 0.88(106/120) |
| Conant et al. | 2019 | 0.19(50/260) | 0.47(122/260) | 0.41(50/122) | NA | NA | NA | NA | NA | NA |
| Shoshan et al. | 2022 | 0.08(417/5182) | 0.16(828/5182) | 0.50(417/828) | NA | NA | NA | NA | NA | NA |

DL Deep learning; NA not available; PPV positive predictive value.

### **Supplementary Table 10.** Detection rate, recall rate, and PPV of the included studies for radiologists with DL assistance.

| **Author** | **Year** | **Unspecified radiologists with DL assistance** | | | **Junior radiologists with DL assistance** | | | **Senior radiologists with DL assistance** | | |
| --- | --- | --- | --- | --- | --- | --- | --- | --- | --- | --- |
|  |  | **Detection rate** | **Recall rate** | **PPV** | **Detection rate** | **Recall rate** | **PPV** | **Detection rate** | **Recall rate** | **PPV** |
| Resch et al. | 2024 | NA | NA | NA | NA | NA | NA | NA | NA | NA |
| Raya-Povedano et al. | 2021 | 0.01(95/15987) | 0.04 (588/15 987) | 0.16(95/588) | NA | NA | NA | NA | NA | NA |
| Park et al. | 2024 | 0.22(57/259) | 0.38(96/256) | 0.59(57/96) | NA | NA | NA | NA | NA | NA |
| Chen et al. | 2025 | NA | NA | NA | NA | NA | NA | NA | NA | NA |
| Chae et al. | 2019 | NA | NA | NA | 0.54(54/100) | 0.64(64/100) | 0.83(54/64) | 0.55(55/100) | 0.65(65/100) | 0.83(55/65) |
| Kim et al. | 2024 | NA | NA | NA | NA | NA | NA | 0.38(90/240) | 0.52(124/240) | 0.73(90/124) |
| Winkel et al. | 2021 | 0.20(49/240) | 0.26(63/240) | 0.78(49/63) | NA | NA | NA | NA | NA | NA |
| Elías-Cabot et al. | 2024 | 0.01(67/6949) | 0.06(396/6949 | 0.17(67/396) | NA | NA | NA | NA | NA | NA |
| Pinto et al. | 2021 | 0.34(64/190) | 0.50(95/190) | 0.67(64/95) | NA | NA | NA | NA | NA | NA |
| Balleyguier et al. | 2017 | 0.23(18/80) | 0.55(44/80) | 0.41(18/44) | NA | NA | NA | NA | NA | NA |
| Bassi et al. | 2023 | NA | NA | NA | 0.51(107/210) | 0.56(117/210) | 0.91(107/117) | 0.50(106/210) | 0.54(113/210) | 0.94(106/113) |
| Conant et al. | 2019 | 0.23(59/260) | 0.67(174/260) | 0.34(59/174) | NA | NA | NA | NA | NA | NA |
| Shoshan et al. | 2022 | 0.08(413/5182) | 0.14(715/5182) | 0.58(413/715) | NA | NA | NA | NA | NA | NA |

DL Deep learning; NA not available; PPV positive predictive value.

### **Supplementary Table 11.** Technical aspects of included studies for DBT.

| **Author** | **Year** | **Manufacturer-Scanner Modality** | **Artificial Intelligence Software** | **Time of acquired DBT images** | **Regions of interest** |
| --- | --- | --- | --- | --- | --- |
| Resch et al. | 2024 | Hologic**-**3Dimensions | ProFound AI 3.0 | Pre-treatments | Fully automatic |
| Raya-Poveda et al. | 2021 | Hologic-Selenia Dimensions | Transpara 1.7.0 | Pre-treatments | Fully automatic |
| Park et al. | 2024 | Hologic-(unspecified model) | ResNet34 | Pre-treatments | Semi-automatic |
| Chen et al. | 2025 | Hologic-Selenia Dimensions | Transpara v1.7.1 | Pre-treatments | Fully automatic |
| Chae et al. | 2019 | GE Healthcare- SenoClaire | ResNe50 | Pre-treatments | Fully automatic |
| Kim et al. | 2024 | Hologic-(unspecified model) | NA | Pre-treatments | Fully automatic |
| Winkel et al. | 2021 | Siemens Healthineers-Mammomat Inspiration | NA | Pre-treatments | Fully automatic |
| Elías-Cabot et al. | 2024 | Hologic-Selenia Dimensions | Transpara 1.7.0 | Pre-treatments | Fully automatic |
| Pinto et al. | 2021 | Siemens Healthcare-MAMMOMAT Inspiration | Transpara 1.6.0 | Pre-treatments | Fully automatic |
| Balleyguier et al. | 2017 | GE Healthcare-SenoClaire | PowerLook Tomo Detection | Pre-treatments | Fully automatic |
| Bassi et al. | 2023 | FUJIFILM-Amulet Innovality | ProFound AI 3.1 | Pre-treatments | Fully automatic |
| Conant et al. | 2019 | Hologic-Selenia Dimensions | PowerLook Tomo Detection 2.0 | Pre-treatments | Manual |
| Shoshan et al. | 2022 | Hologic-Selenia Dimensions | NA | Pre-treatments | Fully automatic |

NA not available.

### **Supplementary Table 12.** DBT-based DL versus radiologists of different levels in the analysis of DBT-based artificial intelligence performance.

| **Subgroup** | **Sample size** | **Number of validation data sets, n** | **Sensitivity(95%C****I)** | **Subgroup difference ^a^** | **Specificity(95%CI)** | **Subgroup difference ^a^** | **AUC (95%CI)** | **Subgroup difference ^a^** |
| --- | --- | --- | --- | --- | --- | --- | --- | --- |
| **DBT-based DL vs. all radiologists** |  |  |  |  |  |  |  |  |
| All radiologists | 12,555 | 8 | 0.83(0.79-0.86) | Z = 0.85, *P* = 0.39 | 0.86 (0.75-0.92) | Z = 0.53, *P* = 0.61 | 0.88 (0.85- 0.91) | Z = 0.46, *P* = 0.64 |
| All radiologists with DL assistance | 36,245 | 14 | 0.85(0.82-0.88) |  | 0.83(0.73-0.89) |  | 0.89(0.86-0.92) |  |
| **DBT-based DL vs. Junior radiologists** |  |  |  |  |  |  |  |  |
| Junior radiologists | 12,555 | 8 | 0.76 (0.66-0.84) | Z = 1.34, *P* = 0.18 | 0.94 (0.48-1.00) | Z = 0.99, *P* = 0.32 | NA |  |
| Junior radiologists with DL assistance | 310 | 2 | 0.84(0.75-0.90) |  | 0.79(0.61-0.90) |  |  |  |
| **DBT-based DL vs. Senior radiologists** |  |  |  |  |  |  |  |  |
| Senior radiologists | 12,555 | 8 | 0.86 (0.81-0.90) | Z = 0, *P* = 1 | 0.93 (0.48-1.00) | Z= 0.88, *P* = 0.38 | 0.90 (0.87- 0.92) | Z= 0.70, *P* = 0.48 |
| Senior radiologists with DL assistance | 961 | 4 | 0.86(0.80-0.91) |  | 0.80(0.64-0.90) |  | 0.93(0.84-1.00) |  |

AUC area under the curve; DL artificial intelligence; NA not available.

^a^ Based on two-sided Z-test.

### **Supplementary Table 13.** DBT-based DL versus radiologists of different levels in the analysis of DBT-based artificial intelligence performance.

| **Subgroup** | **Sample size** | **Number of validation data sets, n** | **Detection rate (95%CI)** | **Subgroup difference ^a^** | **PPV (95%CI)** | **Subgroup difference ^a^** | **Recall (95%CI)** | **Subgroup difference ^a^** |
| --- | --- | --- | --- | --- | --- | --- | --- | --- |
| **DBT-based DL vs. all radiologists** |  |  |  |  |  |  |  |  |
| All radiologists | 12,555 | 8 | 0.15 (0.06-0.30) | Z = 0.52, *P* = 0.603 | 0.62 (0.40- 0.79) | Z = 0.20, *P* = 0.843 | 0.30 (0.19- 0.45) | Z=0.68, *P* = 0.498 |
| All radiologists with DL assistance | 36,245 | 14 | 0.20(0.09-0.38) |  | 0.64(0.46- 0.79) |  | 0.37(0.23- 0.54) |  |
| **DBT-based DL vs. Junior radiologists** |  |  |  |  |  |  |  |  |
| Junior radiologists | 12,555 | 8 | 0.47 (0.42-0.53) | Z = 1.26, *P* = 0.208 | 0.87(0.81-0.91) | Z = 0.55, *P =* 0.579 | 0.55(0.47-0.63) | Z = 0.61, *P* = 0.545 |
| Junior radiologists with DL assistance | 310 | 2 | 0.52(0.46-0.57) |  | 0.89(0.83-0.93) |  | 0.58(0.53-0.64) |  |
| **DBT-based DL vs. Senior radiologists** |  |  |  |  |  |  |  |  |
| Senior radiologists | 12,555 | 8 | 0.36(0.19-0.57) | Z = 1.73, *P* = 0.085 | 0.90(0.70-0.97) | Z= 0.57, *P* = 0.567 | 0.43(0.22-0.68) | Z = 0.99, *P* = 0.320 |
| Senior radiologists with DL assistance | 961 | 4 | 0.64(0.34-0.85) |  | 0.85(0.72-0.93) |  | 0.55(0.50-0.61) |  |

DL artificial intelligence.

^a^ Based on two-sided Z-test.

### **Supplementary Table 14.** Subgroup analysis and meta-regression analysis.

| **Subgroup** | **Number of validation data sets, n** | **Sensitivity(95%CI)** | **Meta-regression *P*-value** | **Specificity(95%CI)** | **Meta-regression *P*-value** |
| --- | --- | --- | --- | --- | --- |
| **Validation** |  |  | 0.61 |  | 0.05 |
| Internal validation | 1 | 0.96 (0.92 - 1.00) |  | 0.40 (0.03 - 0.76) |  |
| External validation | 7 | 0.85 (0.79 - 0.91) |  | 0.78 (0.68 - 0.88) |  |
| **Study design** |  |  | 0.26 |  | 0.47 |
| Retrospective | 7 | 0.93 (0.83 - 1.00) |  | 0.92 (0.85 - 0.99) |  |
| Prospective | 1 | 0.79 (0.53 - 1.00) |  | 0.84 (0.59 - 1.00) |  |
| **Regions of interest** |  |  | 0.64 |  | 0.35 |
| Fully automatic | 6 | 0.86 (0.79 - 0.94) |  | 0.77 (0.63 - 0.91) |  |
| Non-fully automatic | 2 | 0.91 (0.82 - 1.00) |  | 0.65 (0.35 - 0.95) |  |
| **Data splitting method** |  |  | 0.79 |  | 0.29 |
| Independent dataset validation | 6 | 0.83 (0.77 - 0.90) |  | 0.77 (0.64 - 0.91) |  |
| Non-independent dataset validation | 2 | 0.95 (0.91 - 0.99) |  | 0.63 (0.33 - 0.93) |  |
| **Number of patients** |  |  | 0.47 |  | 0.20 |
| < 1000 | 5 | 0.83 (0.72 - 0.90) |  | 0.79 (0.60 - 0.90) |  |
| ≥ 1000 | 3 | 0.93 (0.88 - 0.96) |  | 0.66 (0.43 - 0.83) |  |
| **Region** |  |  | 0.00 |  | 0.21 |
| Europe | 2 | 0.72 (0.55 - 0.89) |  | 0.86 (0.72 - 1.00) |  |
| North America | 4 | 0.91 (0.86 - 0.96) |  | 0.60 (0.41 - 0.80) |  |

### **Supplementary Table 15**. Sensitivity analysis.

| **Analysis Scenario** | **No. of studies** | **Sensitivity (95%CI)** | **Specificity (95%CI)** | **AUC (95%CI)** | **Detection rate** | **PPV** | **Recall** |
| --- | --- | --- | --- | --- | --- | --- | --- |
| Overall | 8 | 0.88 (0.80 – 0.93) | 0.74 (0.59 - 0.85) | 0.89 (0.86 - 0.92) | 0.14 (0.06 – 0.29) | 0.41 (0.18 - 0.70) | 0.39 (0.27 - 0.53) |
| Excluding outliers studies ^a^ | 6 | 0.86 (0.78 - 0.91) | 0.74 (0.62 - 0.83) | 0.88 (0.85 - 0.91) | 0.13 (0.05 - 0.33) | 0.38 (0.14 - 0.69) | 0.36 (0.23 - 0.53) |
| Without low-quality studies ^b^ | 4 | 0.86 (0.77 - 0.91) | 0.80 (0.57 - 0.93) | 0.90 (0.87 - 0.92) | 0.21 (0.15 - 0.27) | 0.61 (0.37 - 0.81) | 0.40 (0.26 - 0.56) |

CI confidence interval; AUC area under the curve; ROC receiver operating characteristic;PPV positive predictive value.

^a^ Outliers and influential points were identified based on the bivariate boxplots. Studies excluded: Pinto et al. 2021, Shoshan et al. 2022.

^b^ Low quality means that the study was rated as having at least one high-risk item in the PROBAST+AI tool (validation sets). Studies excluded: Resch et al 2024, Chen et al. 2025, Bassi et al. 2023, Shoshan et al. 2022.

### **Supplementary Figure 1.** Forest plots of the pooled sensitivity and specificity of deep learning algorithms.


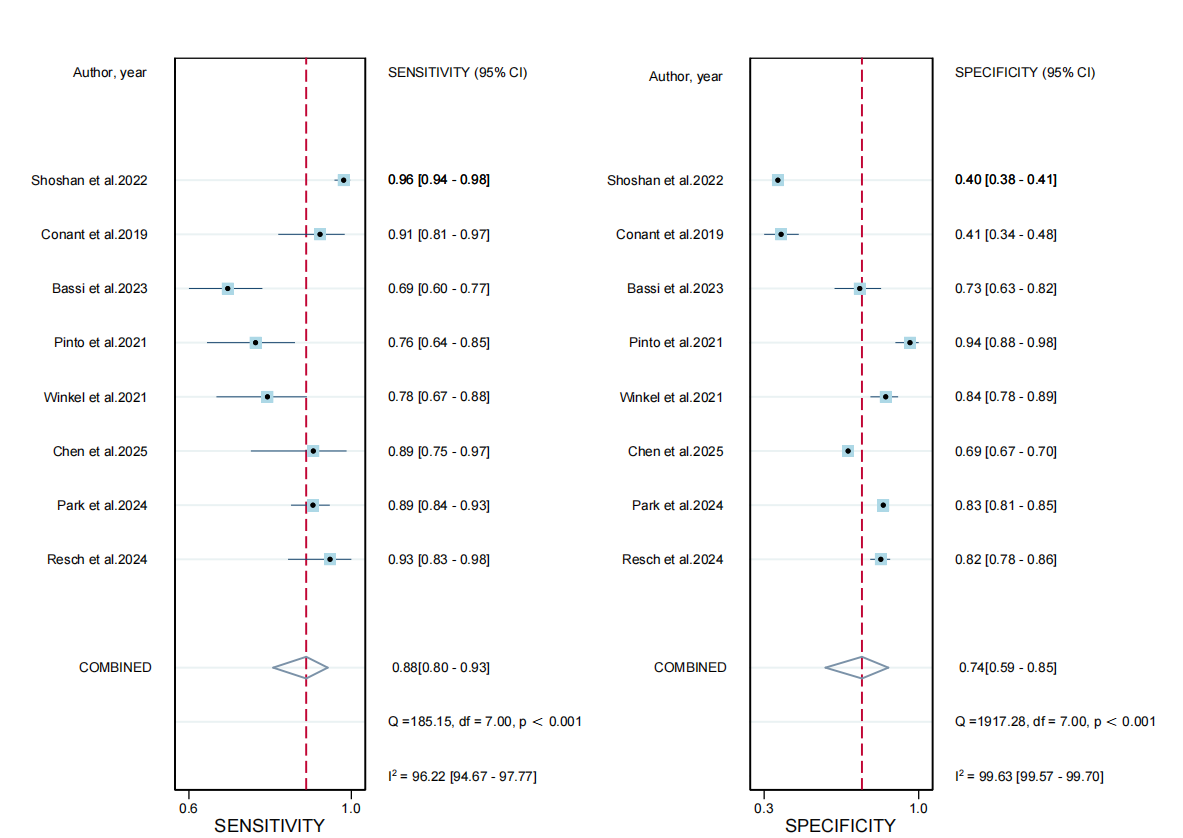


### **Supplementary Figure 2.** Summary receiver operating characteristic (SROC) curve of deep learning algorithms.


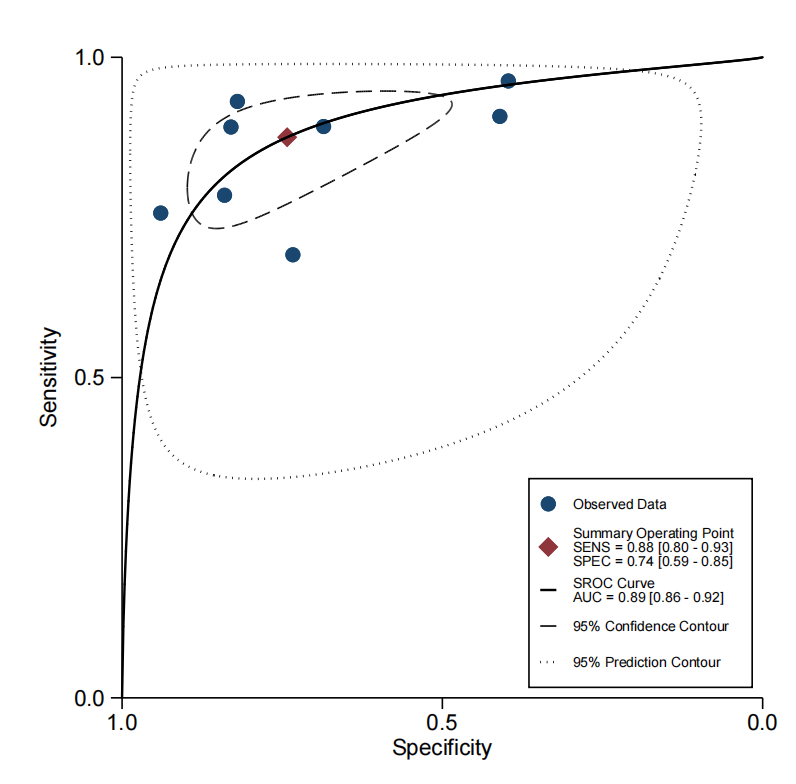


### **Supplementary Figure 3.** Forest plots of the pooled detection rate of deep learning algorithms.


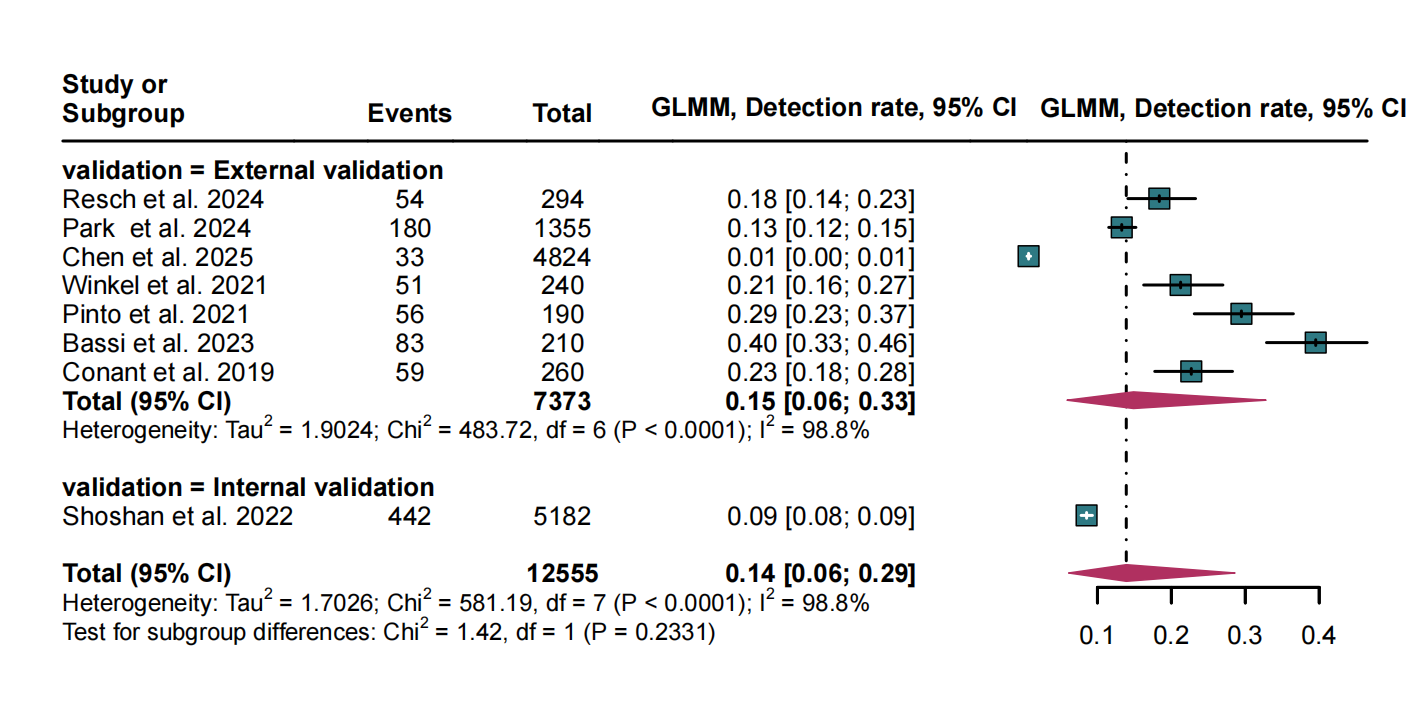


### **Supplementary Figure 4.** Forest plots of the pooled positive predictive value (PPV) of deep learning algorithms.


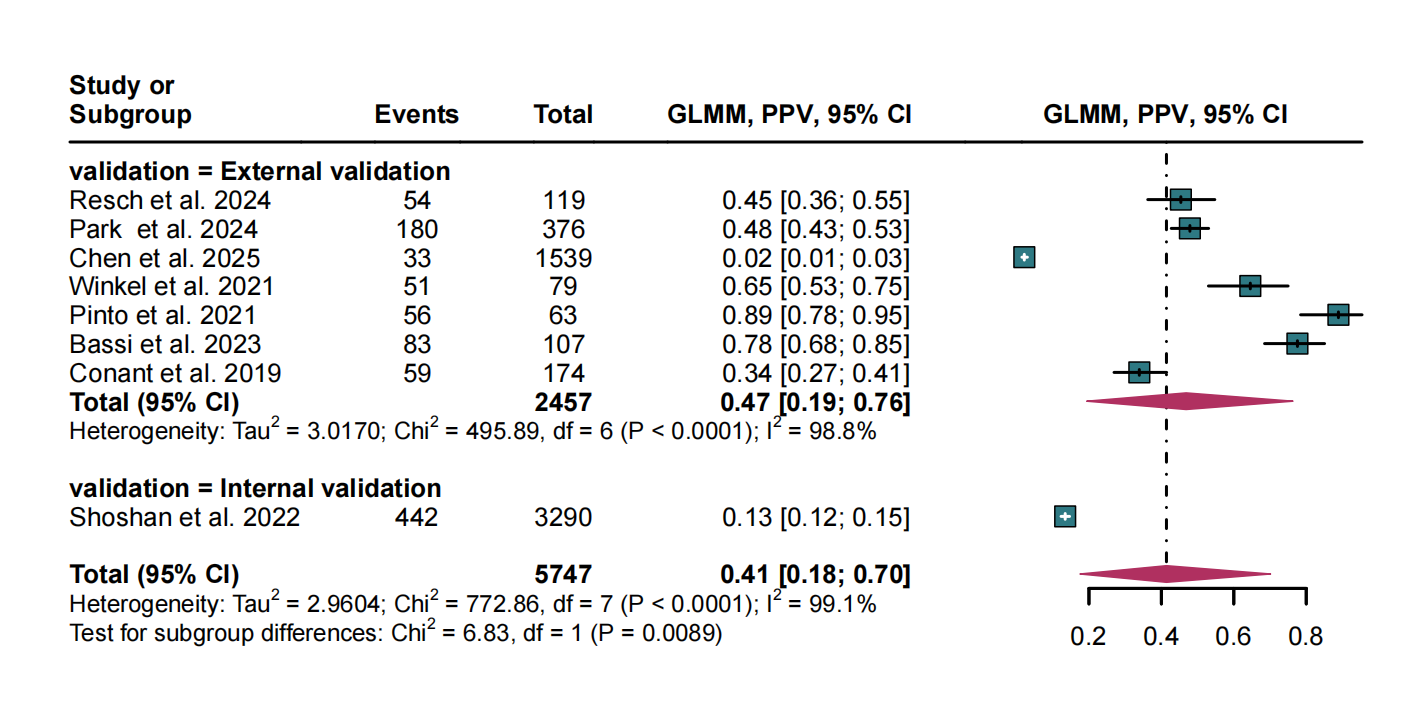


### **Supplementary Figure 5.** Forest plots of the pooled recall rate of deep learning algorithms.


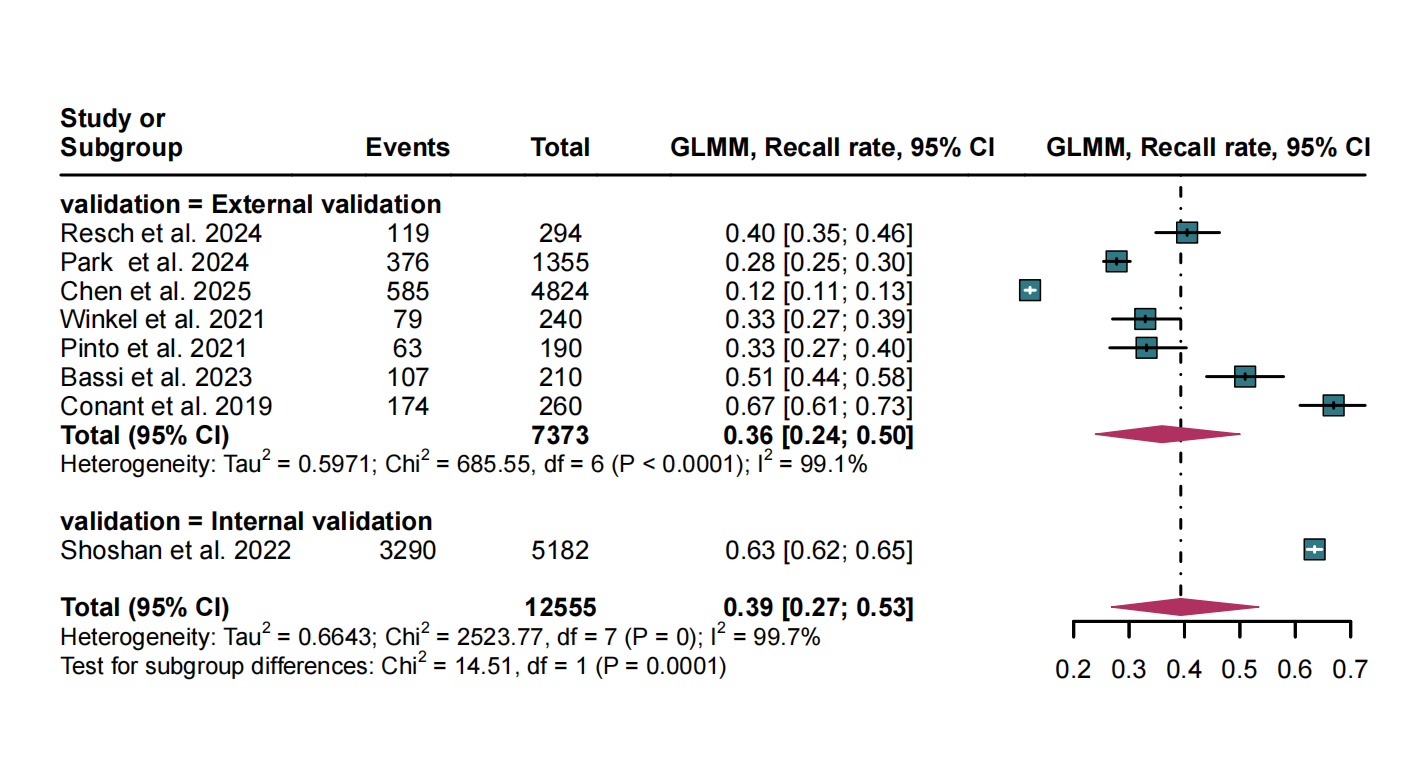


### **Supplementary Figure 6.** Forest plots of the pooled sensitivity and specificity of all radiologists.


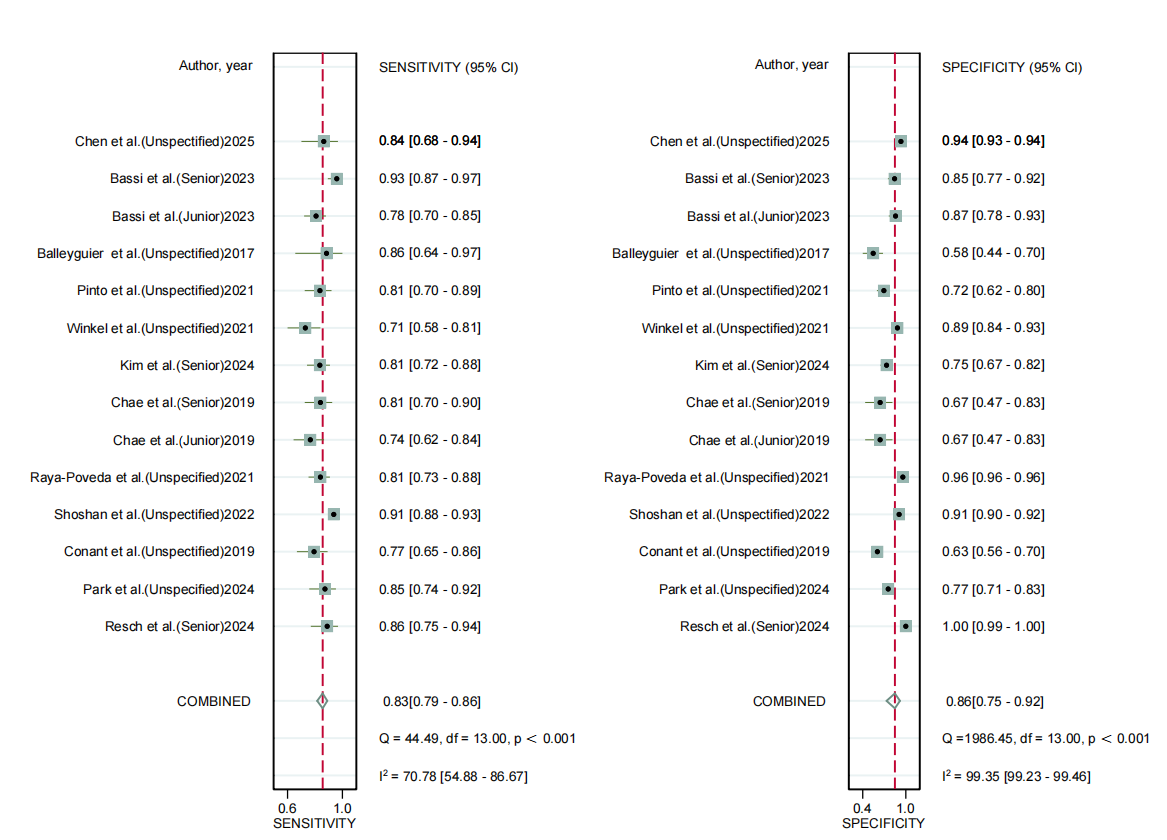


### **Supplementary Figure 7.** Summary receiver operating characteristic (SROC) curve of all radiologists.


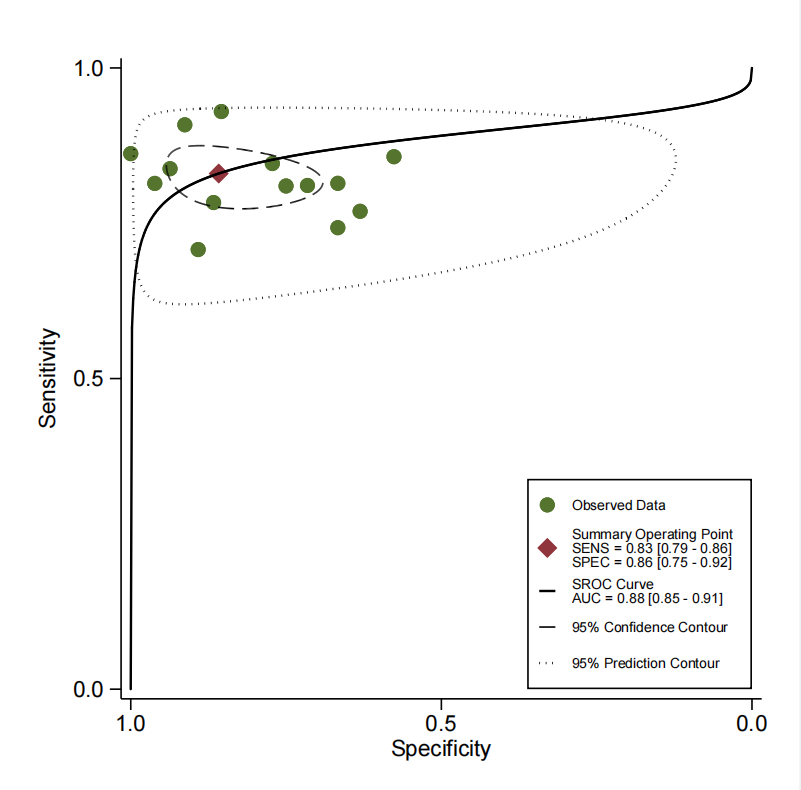


### **Supplementary Figure 8.** Forest plots of the pooled detection rate of all radiologists.


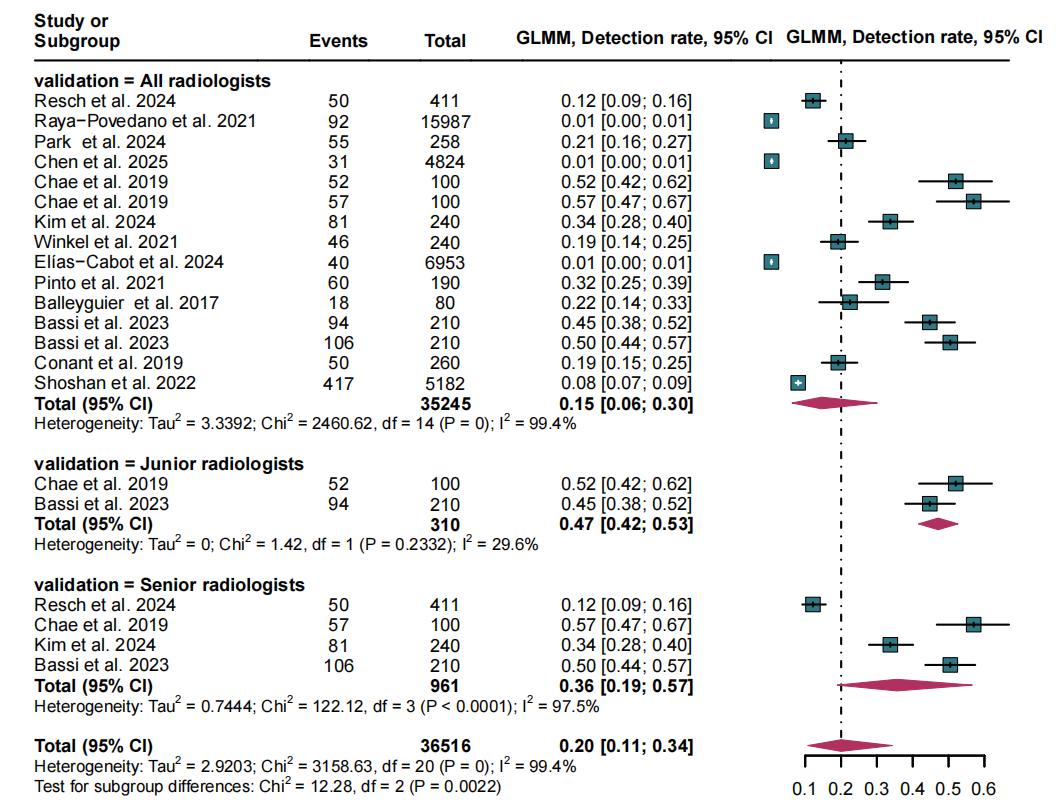


### **Supplementary Figure 9.** Forest plots of the pooled positive predictive value (PPV) of all radiologists.


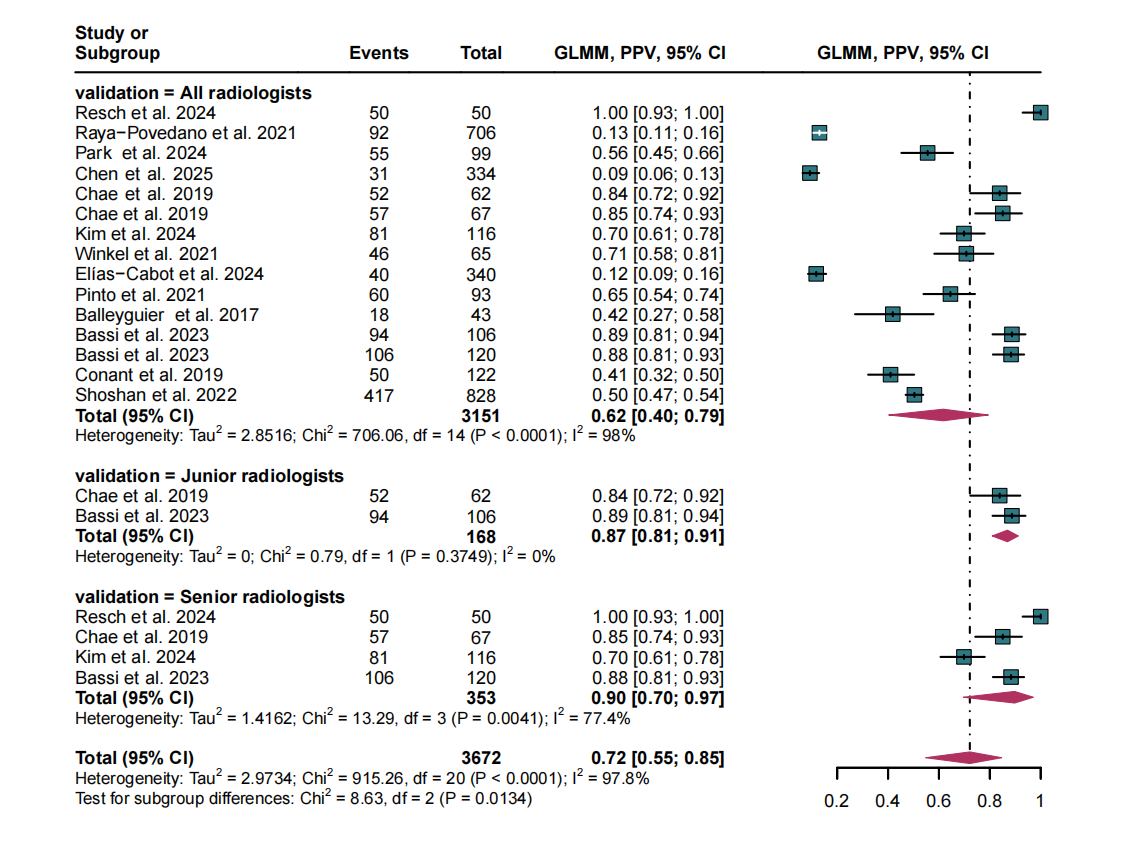


### **Supplementary Figure 10.** Forest plots of the pooled recall rate of all radiologists.


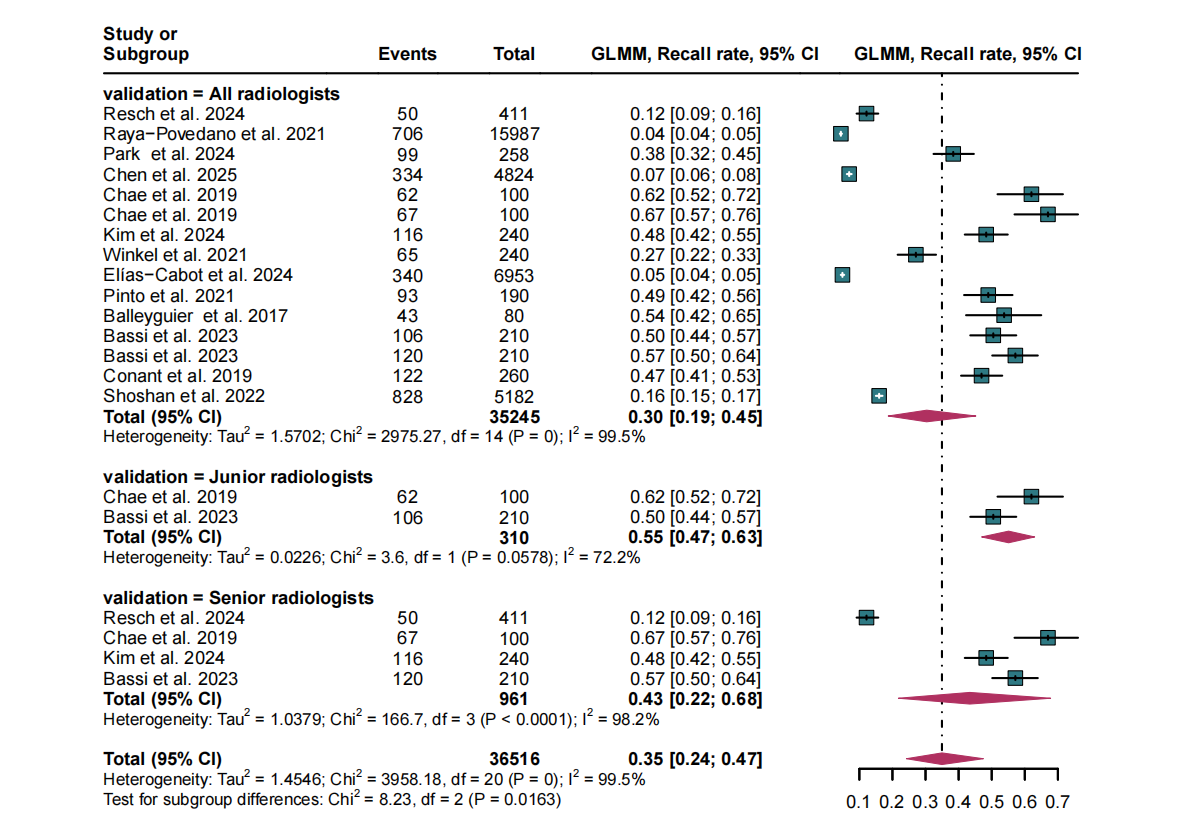


### **Supplementary Figure 11.** Subgroup Analysis Forest plots of the sensitivity and specificity of senior radiologists vs junior radiologists.


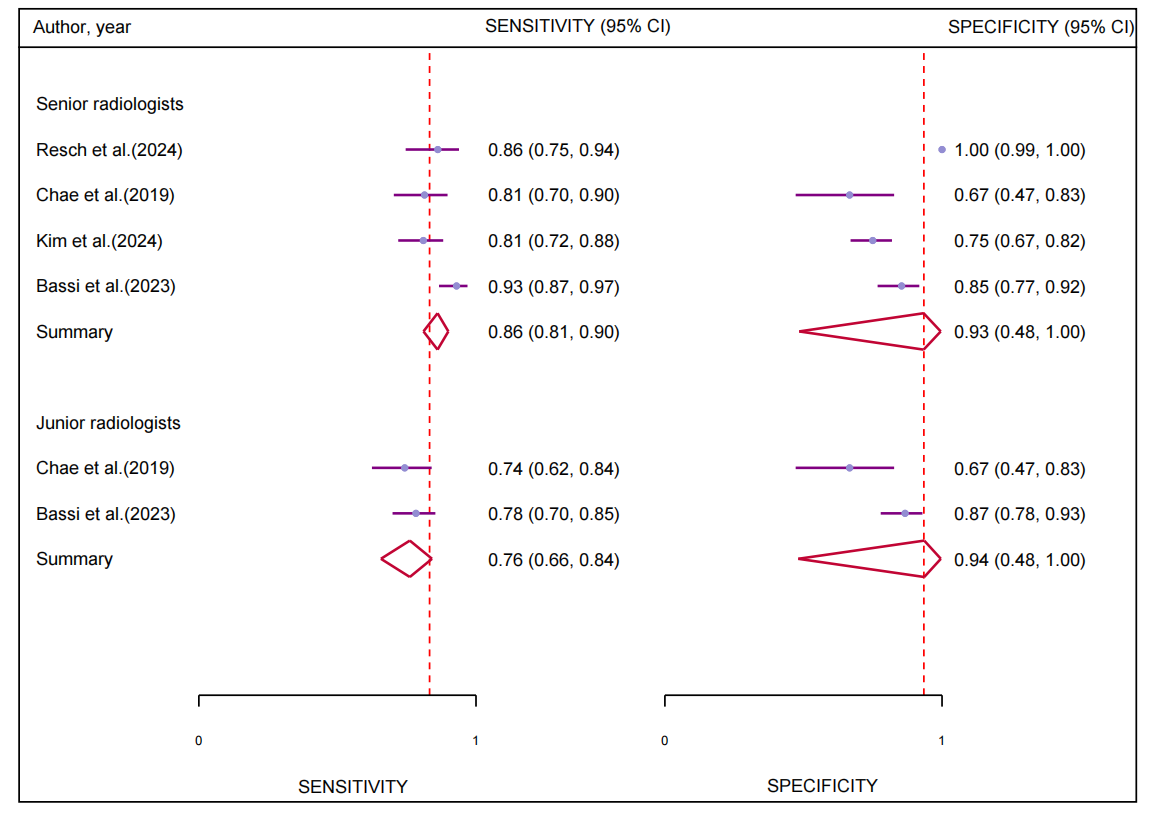


### **Supplementary Figure 12**. Summary receiver operating characteristic (SROC) curve of senior radiologists.


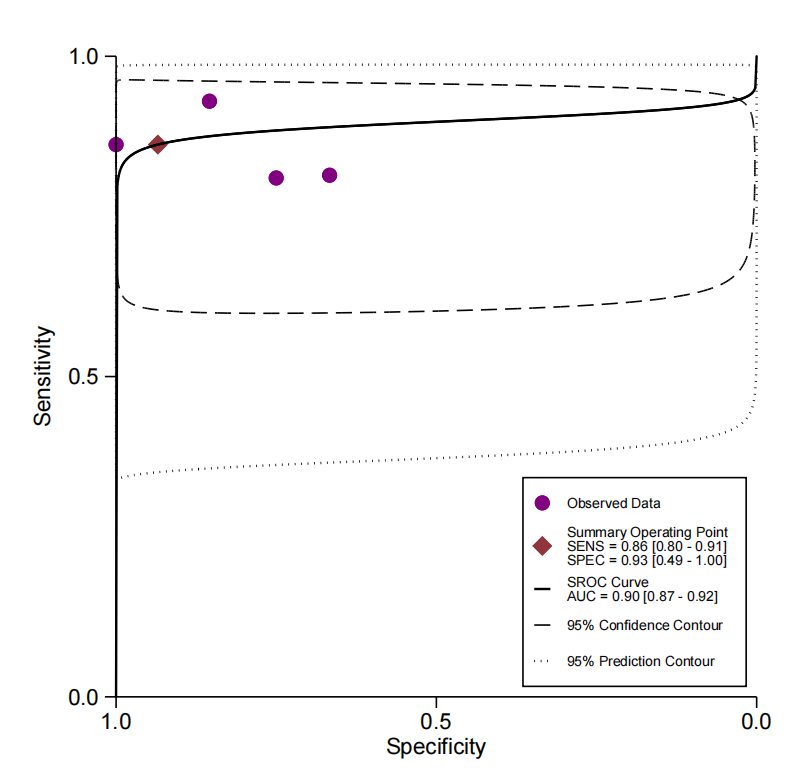


### **Supplementary Figure 13.** Summary receiver operating characteristic (SROC) curve of all radiologists with DL assistance.


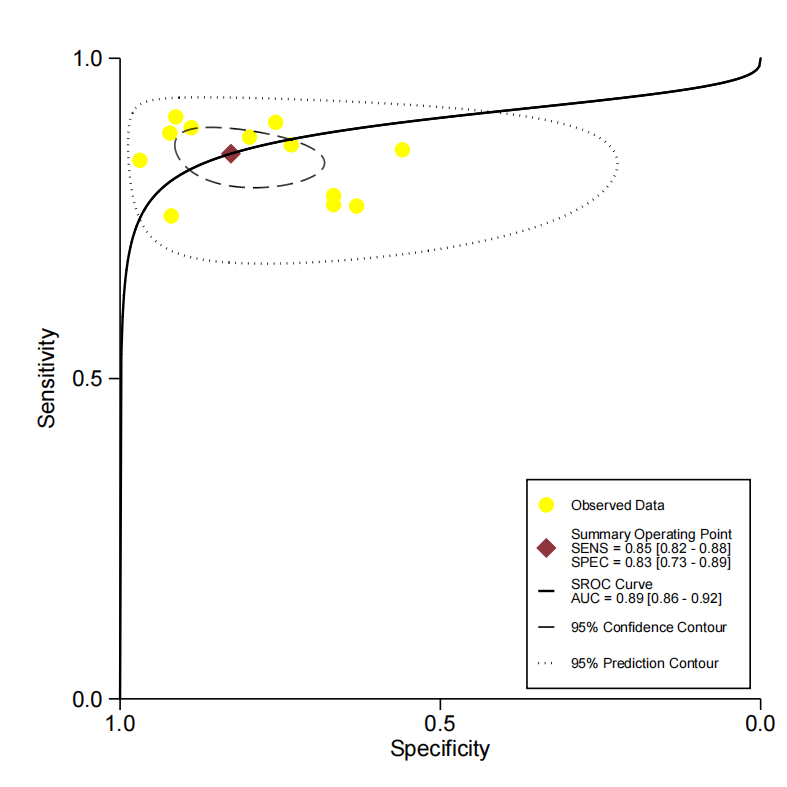


### **Supplementary Figure 14.** Subgroup Analysis Forest plots of the sensitivity and specificity of radiologists with different levels of experience with DL assistance.


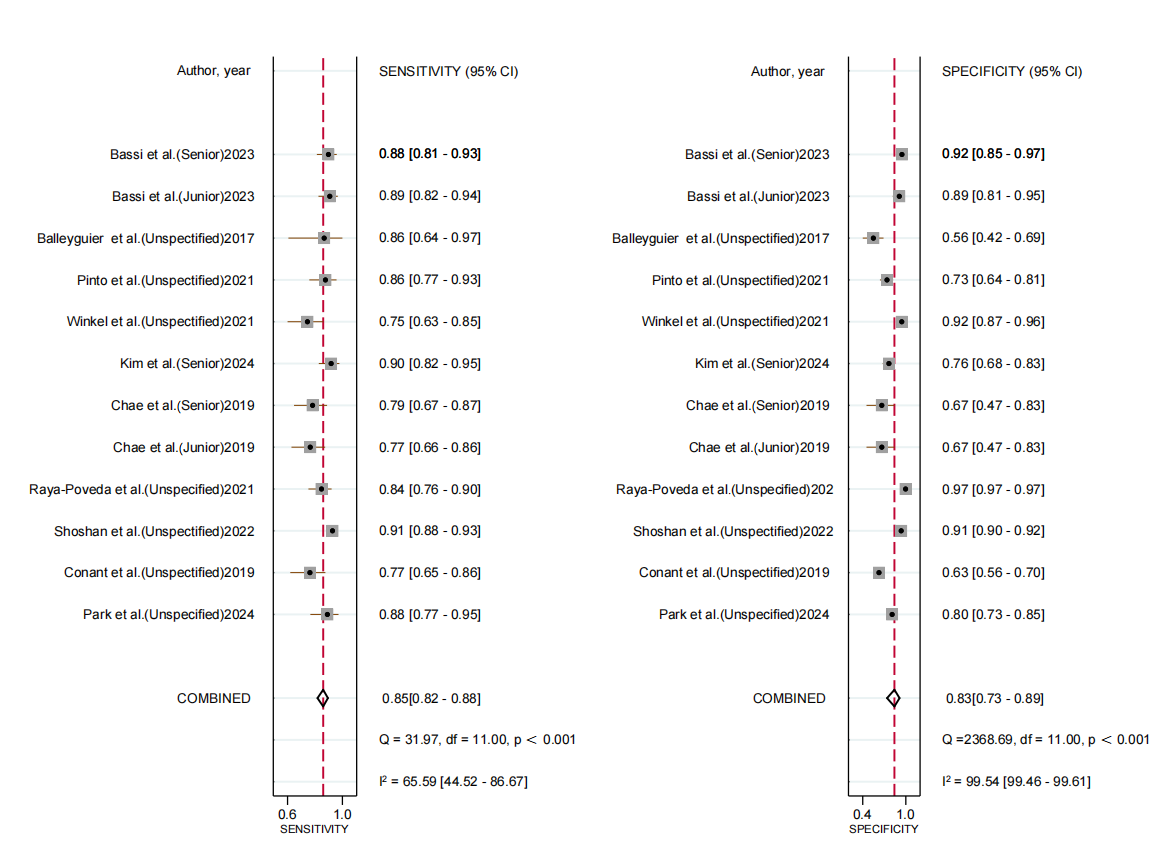


### **Supplementary Figure 15**. Summary receiver operating characteristic (SROC) curve of senior radiologists with DL assistance.


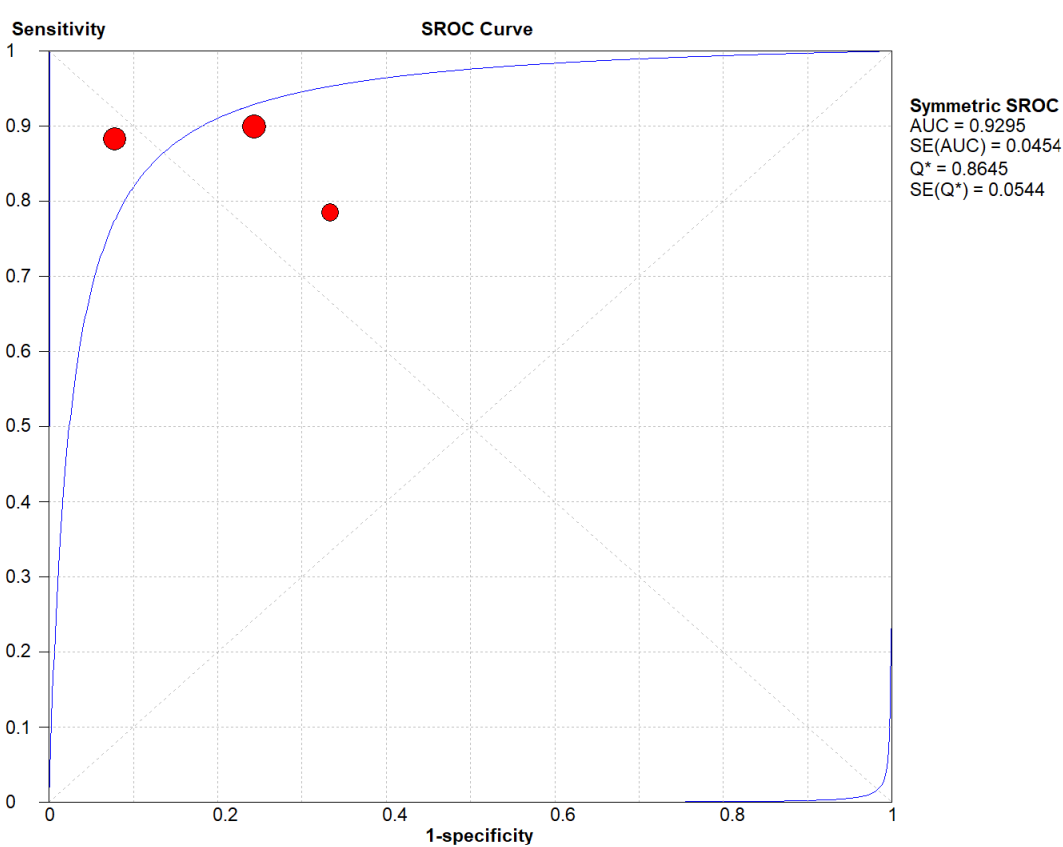


### **Supplementary Figure 16.** Forest plots of the pooled detection rate of all radiologists with DL assistance.


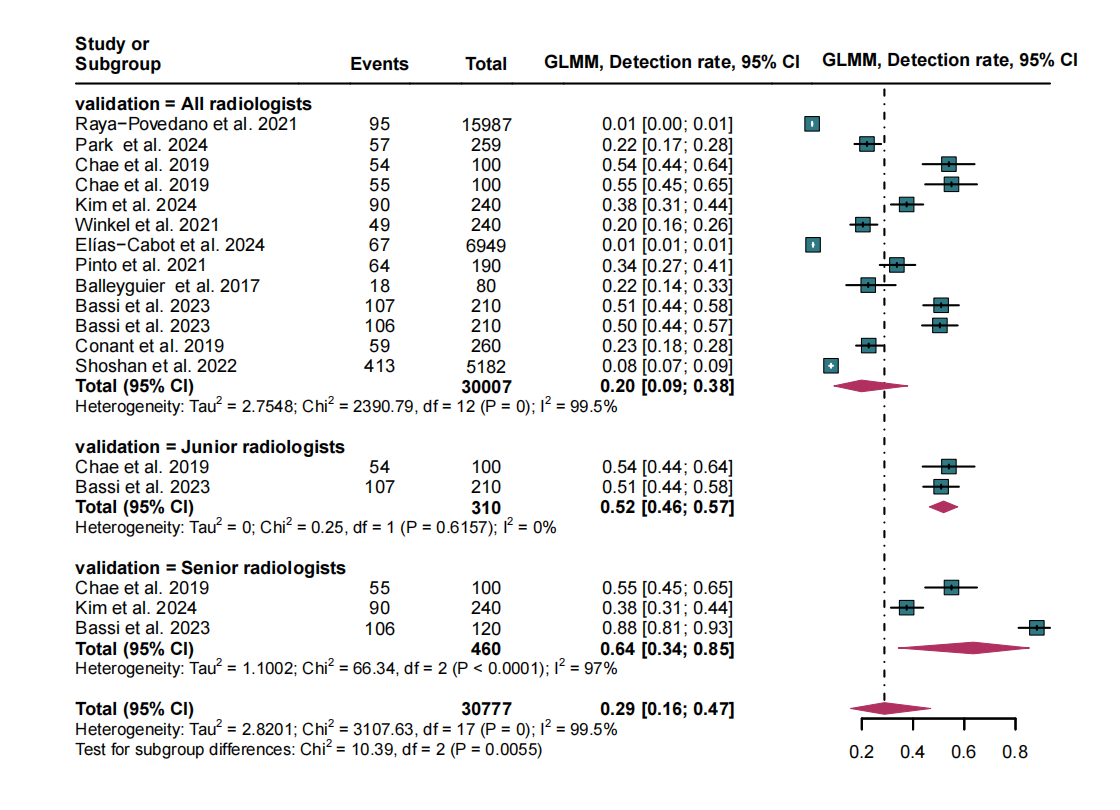


### **Supplementary Figure 17.** Forest plots of the pooled positive predictive value (PPV) of all radiologists with DL assistance.


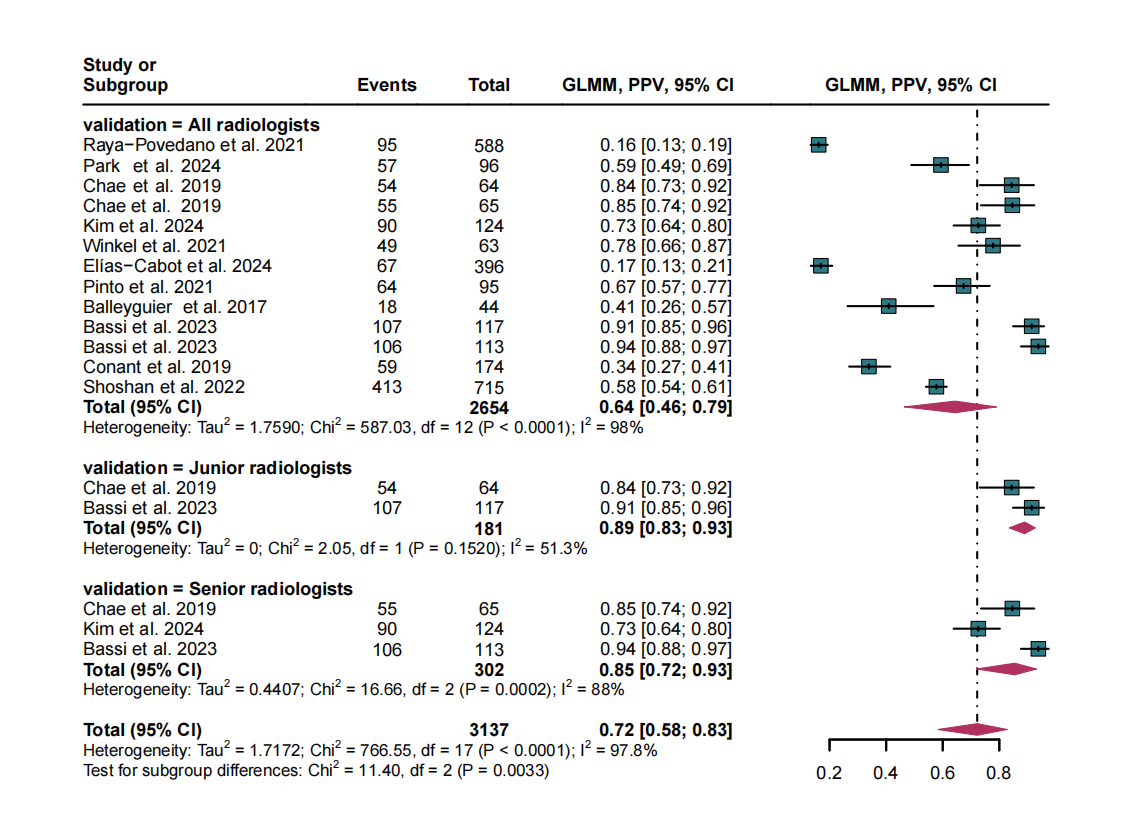


### **Supplementary Figure 18**. Forest plots of the pooled recall rate of all radiologists with DL assistance.


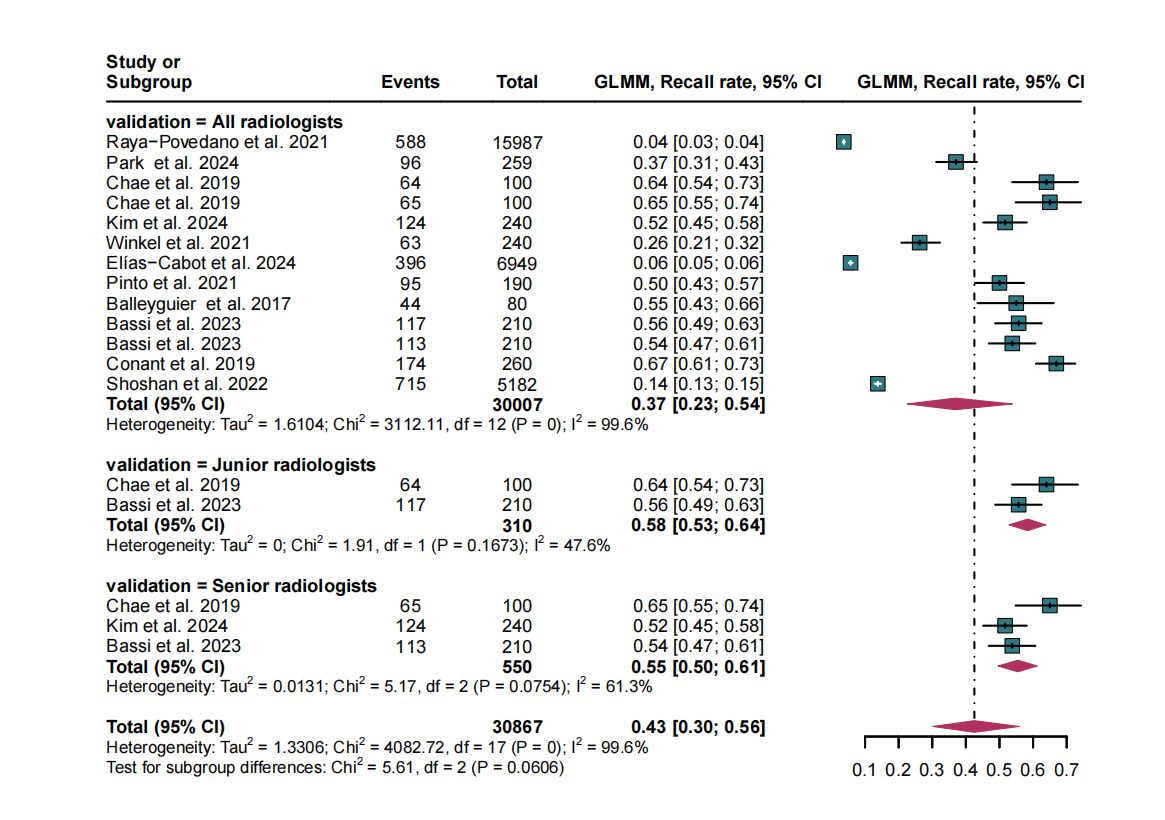

Supplement: Multimedia Appendix 1 [file jmir-v28-e91659-s001.docx]
